# Mapping the intellectual structure of the research of omalizumab in chronic spontaneous urticaria: A bibliometric analysis

**DOI:** 10.1016/j.jacig.2024.100222

**Published:** 2024-02-01

**Authors:** Yuxu Yao, Zhichen Liu, Jiang Ji, Qingqing Jiao

**Affiliations:** a Department of Dermatology, The First Affiliated Hospital of Soochow University, Suzhou, China; b Department of Dermatology, The Second Affiliated Hospital of Soochow University, Suzhou, China; c Department of Ear, Nose, and Throat, The First Affiliated Hospital of Soochow University, Suzhou, China

**Keywords:** Omalizumab, chronic spontaneous urticaria, bibliometric study, VOSviewer, knowledge map

## Abstract

**Background:**

The guidelines for treating chronic spontaneous urticaria (CSU) recommend using the IgE-targeted biologic omalizumab in patients with antihistamine-refractory disease.

**Objective:**

Our aim was to present a bibliometric review of publications related to omalizumab and CSU over the past 2 decades.

**Methods:**

Relevant publications from 2003 to 2022 were extracted from the Science Citation Index-Expanded (SCI-EXPANDED) database in the Web of Science Core Collection database as of January 8, 2023. We utilized CiteSpace (version 6.1.R3), VOSviewer (version 1.6.18), and the R package (version 4.2.1) to analyze and visualize the data. The R package bibliometrix (version 4.2.1) was also used.

**Results:**

Between 2003 and 2022, a total of 566 articles on omalizumab and CSU were published. Since 2014, there has been a rapid increase in publication output. According to the collaboration network, the most influential country, institute, and scholar were the United States, Charité Universitätsmedizin Berlin, and Marcus Maurer, respectively. The study identified the *Journal of Allergy and Clinical Immunology: In Practice* as the most productive journal and the *Journal of Allergy and Clinical Immunology* as the most cocited journal. The analysis of key words revealed the presence of high-frequency terms such as *angioedema, IgE, treatment, anti-IgE, asthma*, and *atopic dermatitis*. Moreover, recent studies in this area have concentrated mainly on biomarkers, dupilumab, and coronavirus 2019 (COVID-19).

**Conclusion:**

There has been a growing interest in the use of omalizumab in CSU in recent years. The current trending topics in this research are the identification of biomarkers and the development of new mAbs for the treatment of CSU.

Chronic spontaneous urticaria (CSU) is a mast cell–driven skin disease characterized by the recurrence of transient wheals, angioedema, or both for more than 6 weeks.^1^ With overall lifetime and point prevalence rates of 1.4% and 0.7%, respectively, CSU affects a significant portion of the global population and is increasing yearly.^2^ Omalizumab is a humanized IgG mAb that binds IgE, which reduces free IgE levels, thereby inhibiting the interaction between IgE and its high-affinity receptor FcεRI and preventing mast cell and basophil activation.^3^ Omalizumab also blocks IgE binding to its low-affinity receptor (CD23) on B cells and antigen-presenting cells. Additionally, omalizumab can dissociate prebound IgE from mast cells and basophils, reducing proximal signaling events and decreasing mast cell mediator release. The treatment of CSU has been virtually revolutionized with the discovery that omalizumab is effective even in the most complex, resistant cases.^4^ Over the years, many observational studies (from 2003 to 2022) were published on the value of omalizumab in the treatment of CSU.

Bibliometrics is the use of statistics to describe or show relationships between published works (eg, books, journal articles, data sets, blogs) and their related metadata (eg, abstracts, key words, citations).^5^^,^^6^ Bibliometrics are now widely used in a variety of medical fields. By analyzing the publication, researchers in related fields can gain a clear understanding of current research trends. However, bibliometrics has not been applied in the study of CSU and omalizumab.

Therefore, we used the Web of Science (WoS) database to perform a bibliometric analysis of articles related to CSU and omalizumab from 2003 to 2022 and utilized multiple bibliometric software tools, namely, VOSviewer,^7^^,^^8^ CiteSpace,^8^^,^^9^ and the R package bibliometrix,^10^ to perform a visualization analysis. We have discussed the trending topics and hotspots in this field and hope that this article will serve as a reference for researchers and drive progress in the research on CSU and omalizumab.

## Methods

### Search strategy

The WoS database (https://www.webofscience.com/wos/woscc/basic-search) was used as the data source. The retrieval time of databases ended in January 2023. The search formula was Topic (TS) = (“chronic spontaneous urticaria”) *OR* TS = (“chronic urticaria”) *OR* TS = (“chronic idiopathic urticaria”) *OR* TS = (“chronic spontaneous urticarial”) *AND* TS = (omalizumab) and language (LA) = (English), and type of document was set to “articles” and “review.” Ethical approval from the Ethics Committee of The Second Affiliated Hospital of Soochow University, Suzhou, China (approval no. JD-LK-2019-105-01) was obtained before the study.

### Data analysis

Taking the respective properties and advantages into consideration, we used VOSviewer, the R package bibliometrix, and CiteSpace at the same time.

VOSviewer (version 1.6.18) was used to extensively explore the relationships within each of the countries and regions, institutions, authors, journals, publications, and author key words.^7^^,^^8^ A circle represents an analytic item, such as a country, institution, journal, or author, in the visual network diagram created by this software. The size of the circle represents the item's quantity. The circles' colors represent various clusters. The thickness of the lines connecting the circles reflects the level of collaboration or cocitation among the analysis items.

The R package bibliometrix (version 4.2.1) (https://www.bibliometrix.org) was used for bibliometric and visualization analysis.^10^ The package bibliometrix is used to create Bradford law diagrams, maps of publications' geographic distribution, trend topic analyses, and Sankey diagrams.

CiteSpace (version 6.1.R3) was used to draw the dual map of journals.^9^ In addition, Microsoft Office Excel 2019 was used to plot the quantitative analysis of publications.

## Results

**Fig 1:**
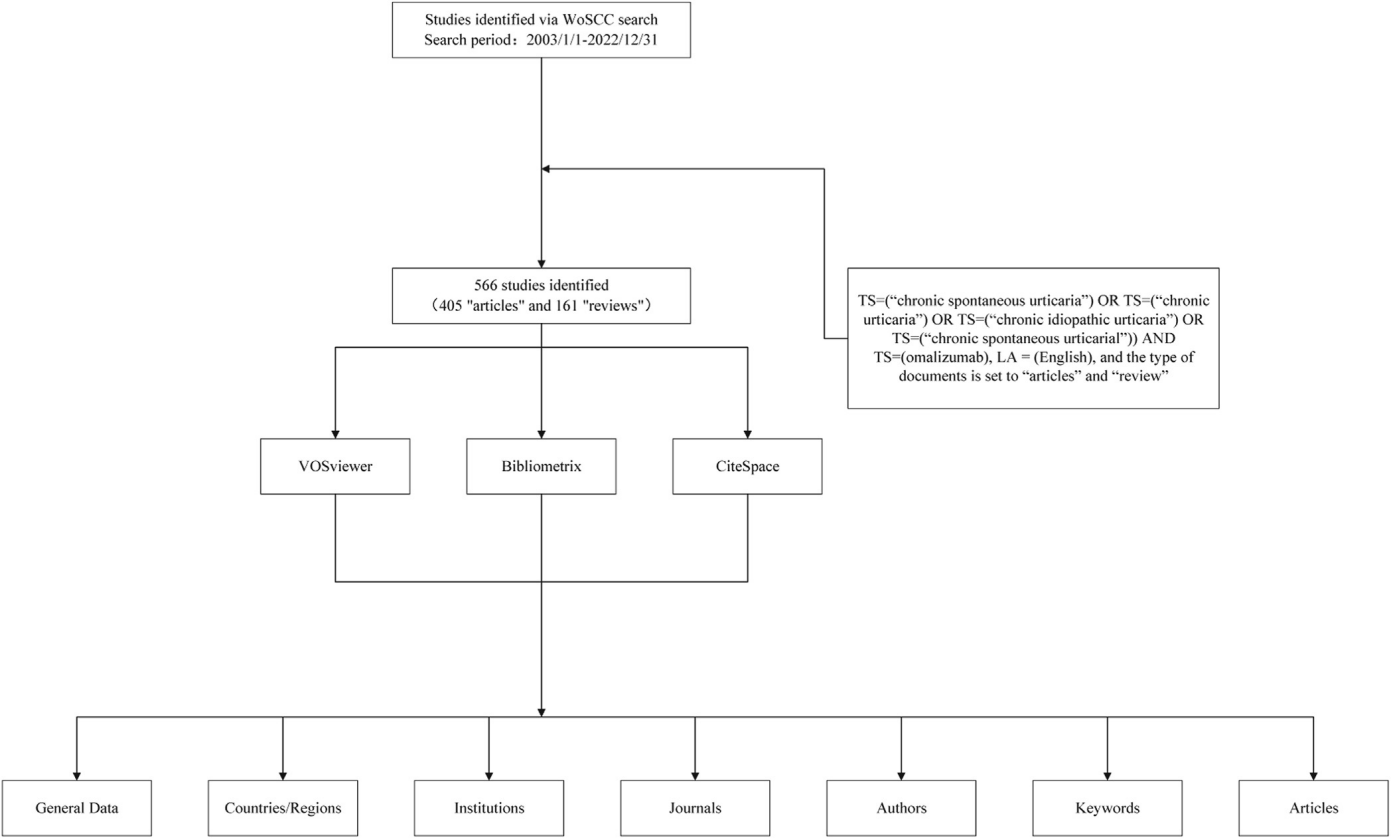
Flowchart of data screening and bibliometric analysis.

There were 566 articles related to the research on use of omalizumab in CSU between January 1, 2003, and December 31, 2022, including 405 articles and 161 reviews. In the past 2 decades, the number of publications on CSU and omalizumab has increased steadily. Fig 1 shows the process of data screening and bibliometric research.

**Fig 2:**
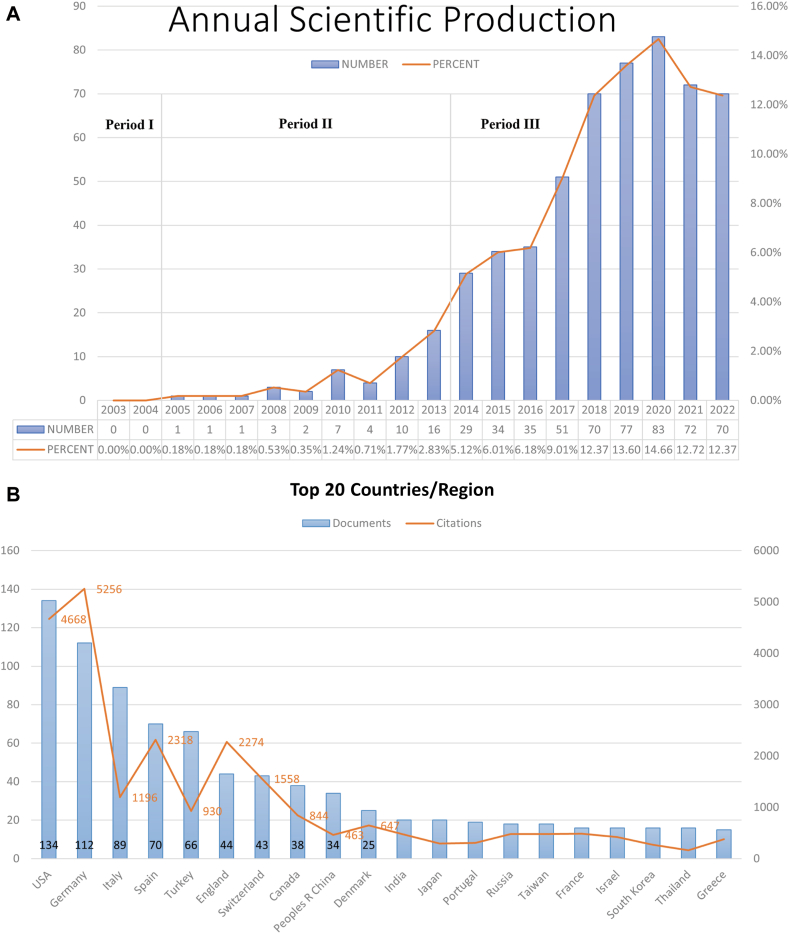

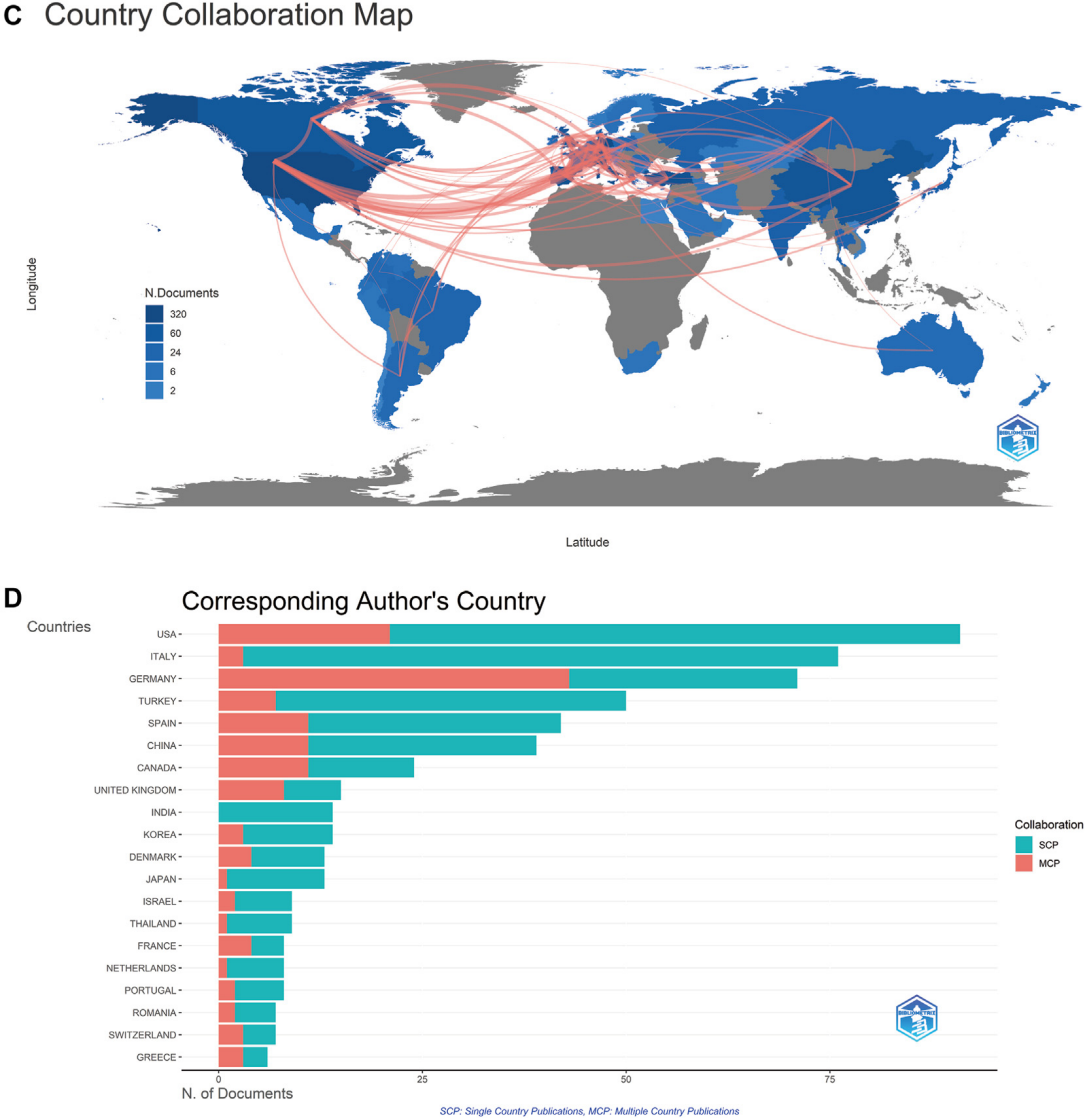

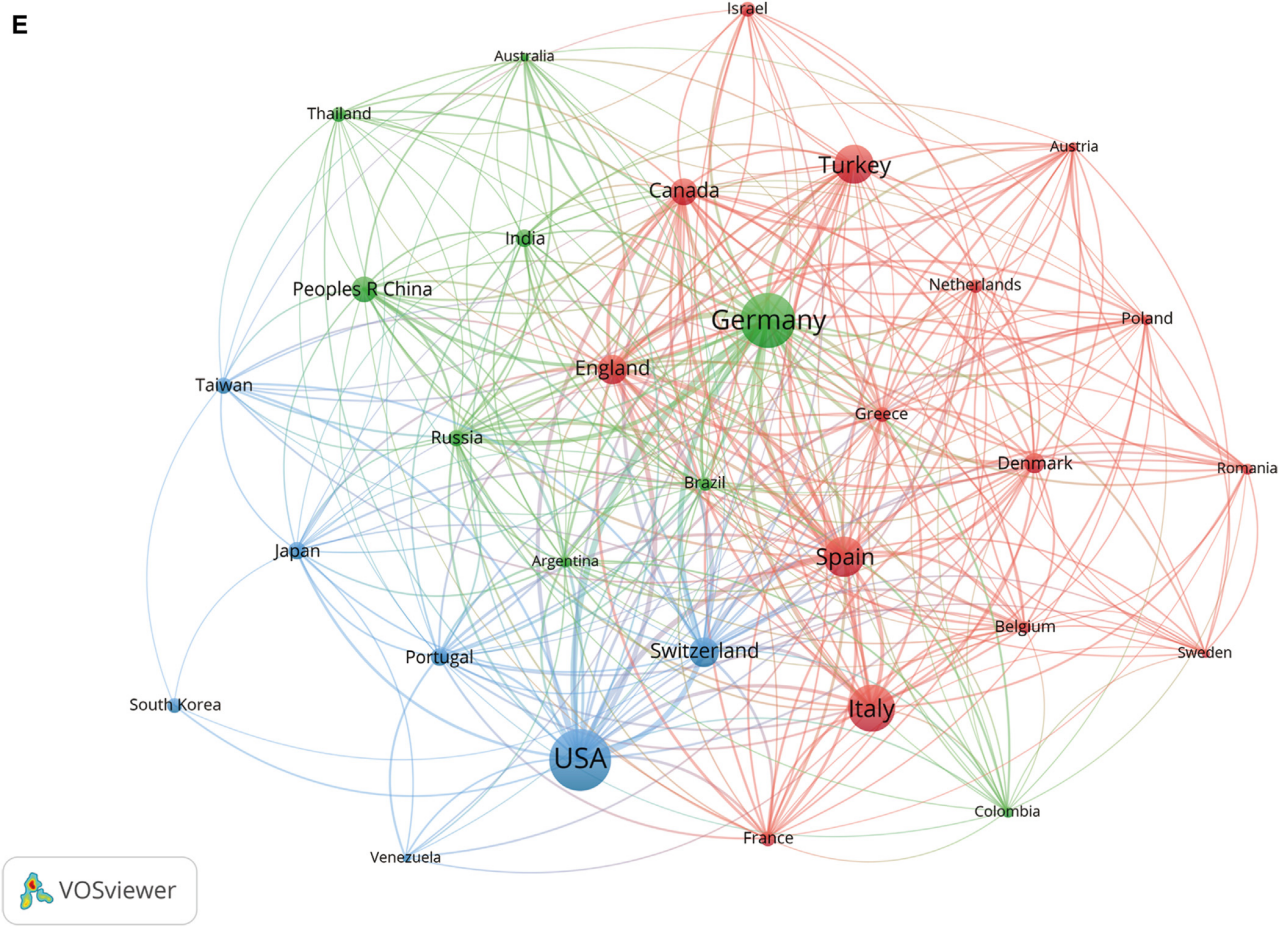

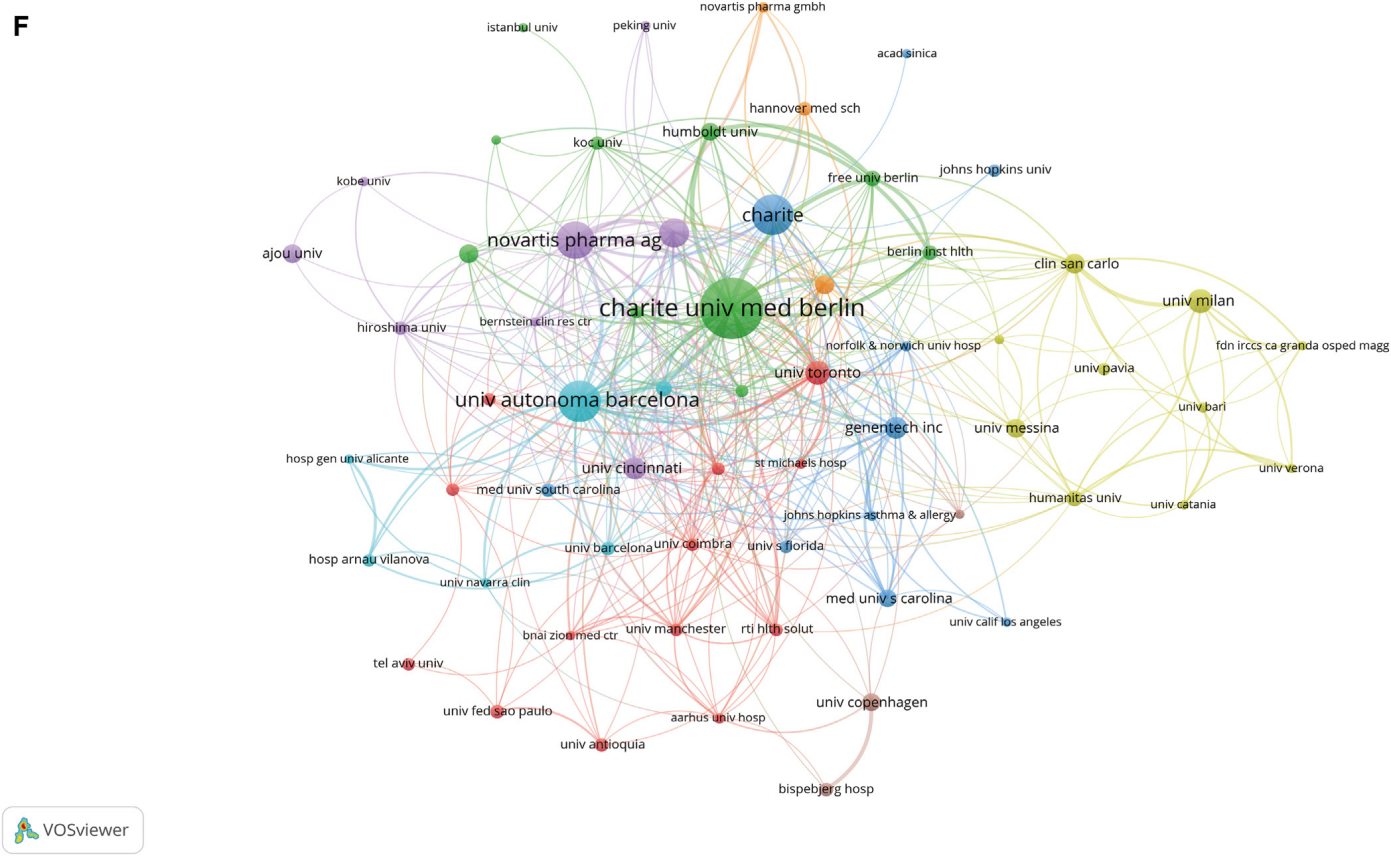
Quantity and trend analysis of published studies and visualization of country/region relationships analysis and institution relationships analysis. **A,** Annual production of scientific research on omalizumab in CSU. **B,** The top 20 countries or regions in terms of number of publications. **C,** The geographic distribution of collaborating countries and regions on the research related to omalizumab in CSU. **D,** Research on omalizumab in CSU by corresponding author’s country. **E** and **F,** The visualization of countries and regions **(E)** and institutions **(F)** involved in research on omalizumab in CSU. *MCP*,  Multiple-country production; *SCP*, single-country production.

As shown in Fig 2, *A*, judging from the rate of growth of the number of publications each year, the whole period can be divided into 3 parts: period I (2003-2004), period II (2005-2013), and period III (2014-2022). Before 2005, there was no relevant research in this field.

### Countries and institutions

The 566 articles came from 60 countries and 1036 institutions. As shown in Fig 2, *B*, the United States had the highest number of publications (n = 134), followed by Germany (n = 112), Italy (n = 89), and Spain (n = 70). Meanwhile, the United States had the highest number of corresponding authors, followed by Italy (Fig 2, *D*).

Subsequently, we filtered and visualized the 50 countries based on having at least 5 publications and constructed a collaborative network based on the number of publications and relationships in each country. Notably, there were many active collaborations between different countries (Fig 2, *C*). For instance, the United States cooperated closely with Germany, Switzerland, Spain, and the United Kingdom, and Germany actively cooperated with the United Kingdom, Spain, and Switzerland (Fig 2, *E*). Fig 2, *F* showed the VOSviewer results for visualizing interinstitutional collaborations, which showed that 63 institutions with more than 5 publications and more than 50 citations were selected for analysis. Charité Universitätsmedizin Berlin, Germany, had the strongest collaboration, with a total link strength (TLS) of 158, followed by Universitat Autònoma de Barcelona, Spain (TLS = 115), and Novartis Pharma AG, Switzerland (TLS = 75).

Meanwhile, Table I shows the 10 institutions with the most publications, with Charité Universitätsmedizin Berlin publishing the most (n = 56), followed by Universitat Autònoma de Barcelona (n = 34). Of the 10 institutions, 7 are in Europe and 3 are in South America. It is worth noting that the first-ranked Charite Univ Med Berlin and the third-ranked charite both refer to Charité - Universitätsmedizin Berlin, which are different names for the same institution. The fourth-ranked Novartis Pharma AG and fifth-ranked Novartis Pharmaceut are different divisions of the same company, both of which are part of Novartis AG.

**Table I:** The 10 most productive institutions in terms of research on use of omalizumab in CSU

Abbreviated institution name	Full institution name	TLS	No. of documents	No. of citations
Charite Univ Med Berlin	Charité - Universitätsmedizin Berlin	158	56	1882
Univ Autonoma Barcelona	Universitat Autònoma de Barcelona	115	34	1667
Charite	Charité - Universitätsmedizin Berlin	46	33	2789
Novartis Pharma AG	Novartis Pharma AG	75	29	915
Novartis Pharmaceut	Novartis Pharmaceuticals	50	21	1201
Univ Toronto	University of Toronto	66	17	478
Univ Milan	University of Milan	27	17	330
Genentech Inc	Genentech, Inc	46	15	1890
Univ Cincinnati	University of Cincinnati	44	15	444
Clin San Carlo	Clinica San Carlo	41	13	311

Both Charite Univ Med Berlin (ranked first) and Charite (ranked third) refer to Charité - Universitätsmedizin Berlin and, thus, are different names for the same institution. Novartis Pharma AG (ranked sixth) and Novartis Pharmaceut (ranked seventh) are different divisions of the same company (ie, Novartis AG).

### Authors and cocited authors

**Fig 3:**
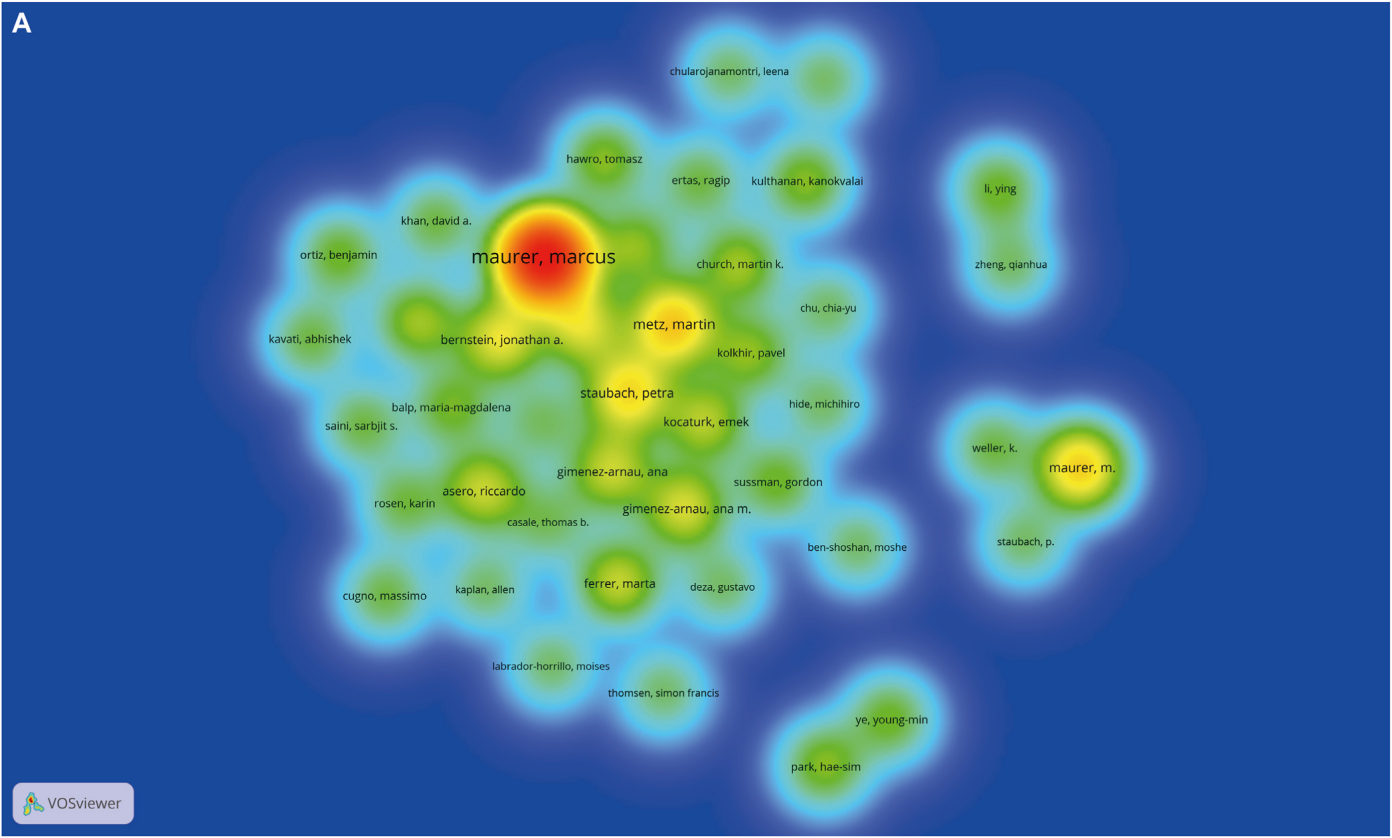

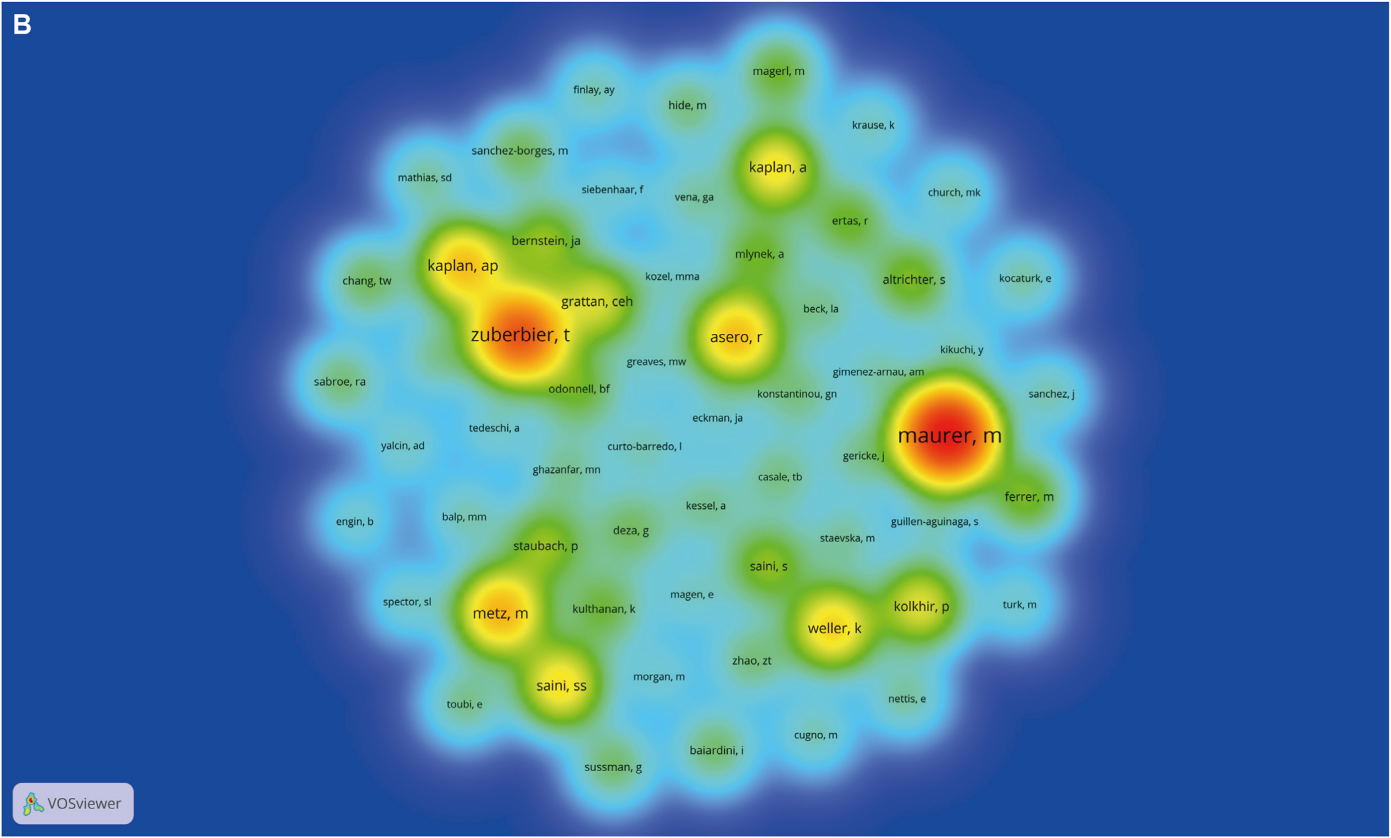

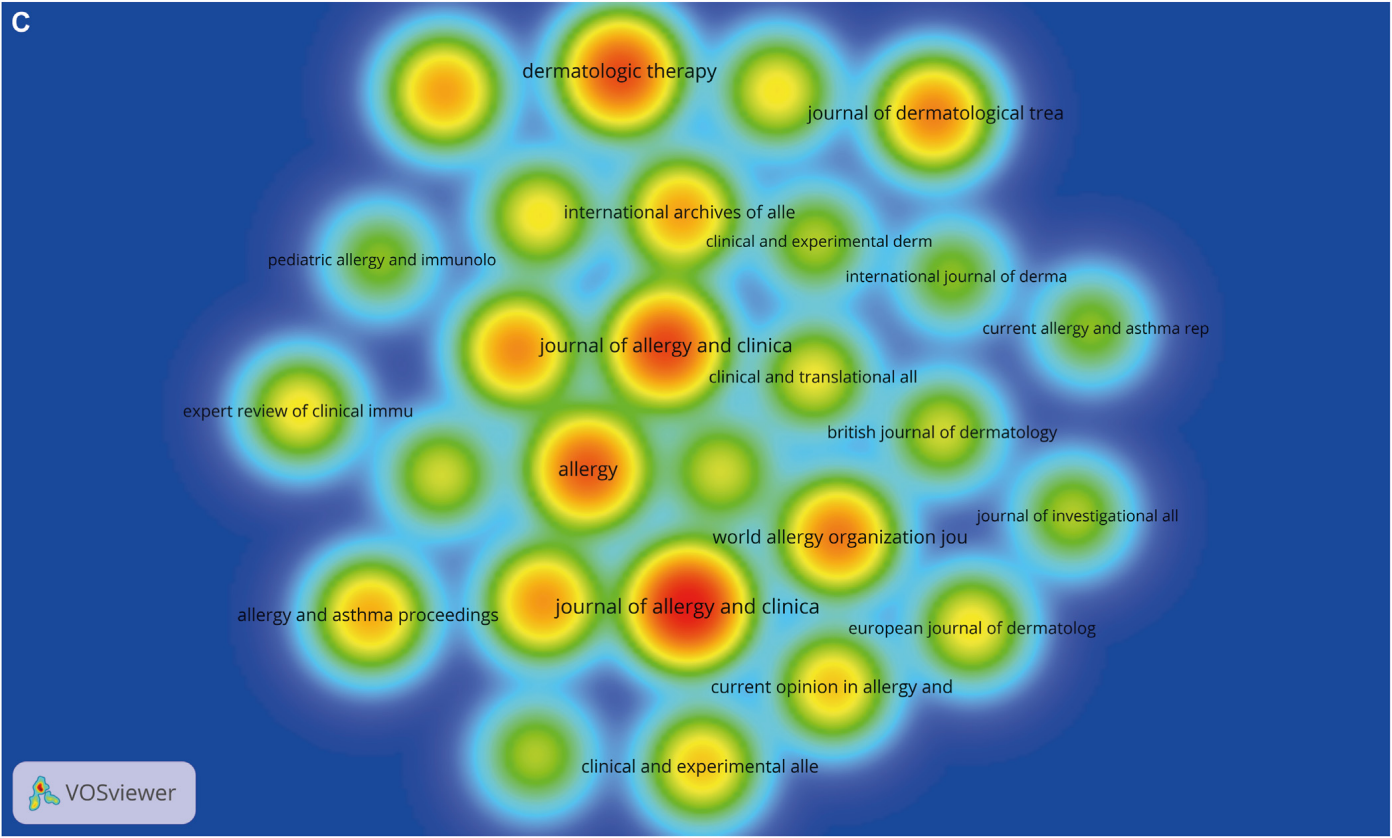

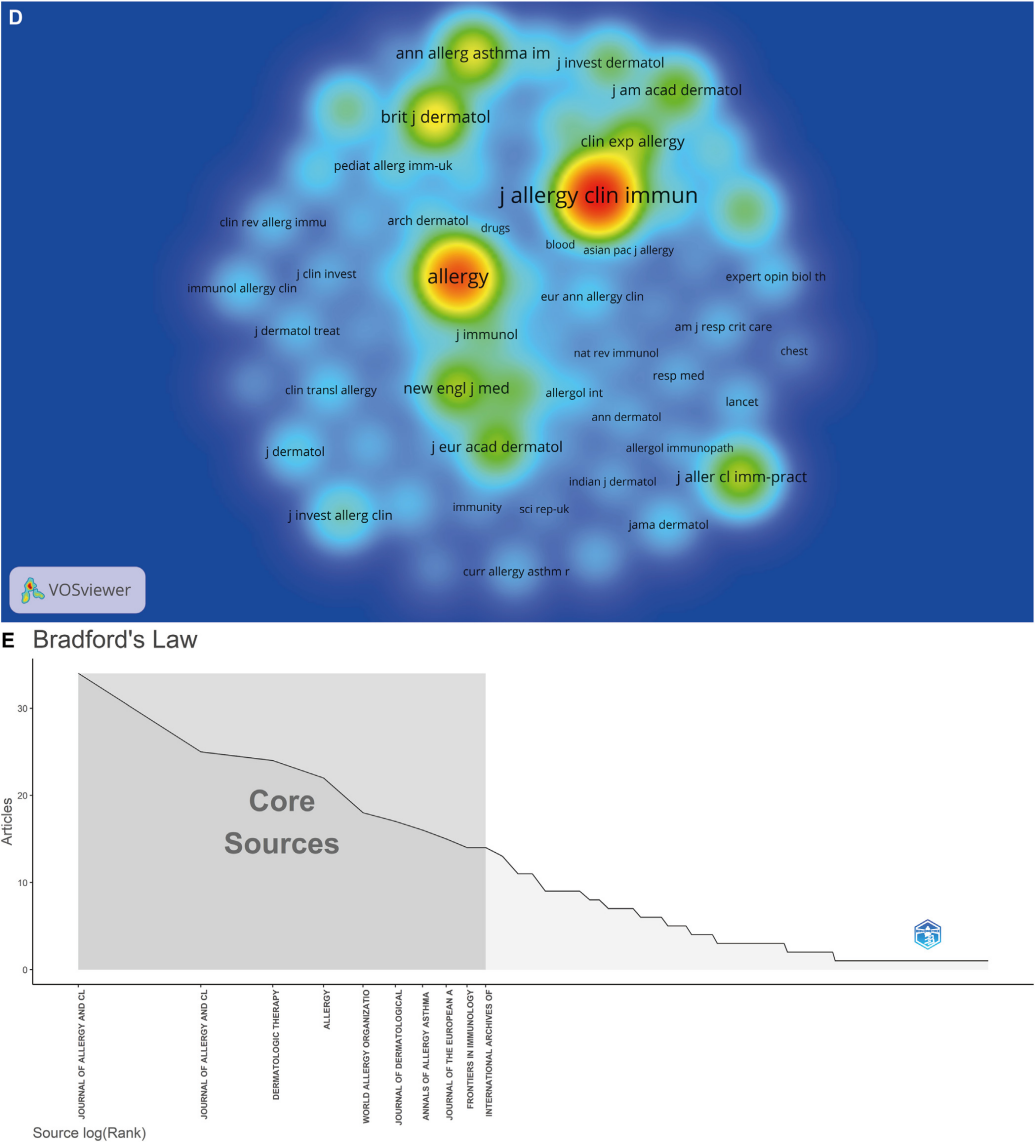
Visualization of analysis of author relationships and analysis of journal relationships. VOSviewer visualization of authors (**A**), cocited authors (**B**), journals **C**), and cocited journals **D**) in research on omalizumab in CSU. **E,** The core journals in terms of research on omalizumab in CSU based on the Bradford law. **A** and **C**, One node represents an author (**A**) or journal (**C**). The node's size reflects the frequency of author's or journal’s cooccurrence, and the node's color reflects the author's or journal’s cooccurrence intensity. **B** and **D**, Size of the nodes reflects the citation frequency of the authors (**B**) and journals (**D**). Color of the nodes reflects the citation strength of the authors or journals, and nodes' labels reflect the authors' or journal’s names.

A visual analysis of the authors in the literature revealed 2303 authors of 566 articles, with the most published author being Marcus Maurer, who published a total of 63 articles. Through calculation by the Price law,^11^ m (the lowest number of papers published by high-yield scientists) = 5.95, and 43 core authors with at least 6 publications were counted. Then, with the help of VOSviewer, an author density graph was generated, as shown in Fig 3, *A*. Meanwhile, Fig 3, *B* shows the different authors' citation frequency, strength, and citation time. It should be noted that specific authors may have different spellings of their name; for example, in terms of publication volume, Maurer M and Marcus Maurer were ranked third and first, respectively, even though they refer to the same person. Therefore, author names were harmonized, and a revised ranking of author co-occurrences was produced, from which authors with a publication count equaling or exceeding 10 were selected for inclusion in Table II.

**Table II:** Authors and cocited authors

Top 10 authors in terms of number of publications			Top 10 authors in terms of number of cocitations		
Author	Paraphrase	No. of documents	Cocited authors	Paraphrase	No. of cocitations
Maurer, Marcus	Maurer, Marcus	82	Maurer, M	Maurer, Marcus	1058
Gimenez-Arnau, Ana M.	Gimenez-Arnau, Ana M.	25	Zuberbier, T	Zuberbier, Torsten	694
Metz, Martin	Metz, Martin	20	Metz, M	Metz, Martin	385
Staubach, Petra	Staubach, Petra	17	Kaplan, Ap	Kaplan, Allen P.	355
Bernstein, Jonathan A.	Bernstein, Jonathan A.	14	Asero, R	Asero, Riccardo	353
Zuberbier, Torsten	Zuberbier, Torsten	13	Weller, K	Weller, Karsten	306
Asero, Riccardo	Asero, Riccardo	12	Saini, SS	Saini, Sarbjit S.	282
Ferrer, Marta	Ferrer, Marta	12	Kaplan, A	Kaplan, Allen	271
Kocaturk, Emek	Kocaturk, Emek	11	Kolkhir, P	Kolkhir, Pavel	229
Church, Martin K.	Church, Martin K.	10	Grattan, CEH	Grattan, Clive E.H.	213
Kaplan, Allen P.	Kaplan, Allen P.	10			

Columns 1 to 3, which deal with the top 10 authors in terms of publication volume include 11 individuals, with the last 2 authors tied for the 10th spot. Columns 1 to 6 present corrected raw data that have not undergone name normalization processing.

After analyzing Fig 3, *A* and *B* and Table II, we concluded that Marcus Maurer was the dominant figure in the field, as evidenced by the author density graph and cocitation author density graph. Additionally, Marcus Maurer had the highest number of publications (n = 82) and cocitations (n = 1058), making him the leading expert in the field. The second highest number of publications was attributed to Ana M. Gimenez-Arnau (n = 25). Martin Metz was in third place in terms of number of publications (n = 20) (Table II).

Regarding cocitations, Torsten Zuberbier and Martin Metz ranked second and third, with 694 and 385 publications, respectively (Table II). Notably, with the exception of Ana M. Gimenez-Arnau from the Universitat Autònoma de Barcelona, all 3 core authors mentioned in this study were affiliated with Charité Universitätsmedizin Berlin.

### Journals and cocited journals

A total of 171 journals were involved in this field of study. We filtered the journals with fewer than 5 publications and fewer than 50 citations and constructed a density view of the 27 journals obtained (Fig 3, *C*). Cocited journals are journals that are cited together in a set of articles. Likewise, to build the density view, from the 1980 cocited journals, we selected those that were cited more than 50 times (Fig 3, *D*).

As shown in Table III, the *Journal of Allergy and Clinical Immunology: In Practice* (n = 34) was identified as the most productive journal in this research area, followed by the *Journal of Allergy and Clinical Immunology* (n = 25) and *Dermatologic Therapy* (n = 24), all of which are American journals. In terms of cocited journals, the *Journal of Allergy and Clinical Immunology* (n = 4088) was the most cocited journal, followed by *Allergy: European Journal of Allergy and Clinical Immunology* (n = 2796) and the *British Journal of Dermatology* (n = 1057), both of which are European journals. Of the top 10 journal articles published, 4 were affiliated with North American institutions (all from the United States), 4 were affiliated with European institutions, and 2 were international journals. Among the top 10 cocited journals, 6 were US journals and 4 were European.

**Table III:** Journals and cocited journals

Top 10 journals		Top 10 cocited journals	
Journals	No. of documents	Cocited journals	No. of cocitations
*Journal of Allergy and Clinical Immunology: In Practice*	34	*Journal of Allergy and Clinical Immunology*	4088
*Journal of Allergy and Clinical Immunology*	25	*Allergy*	2796
*Dermatologic Therapy*	24	*British Journal of Dermatology*	1057
*Allergy*	22	*Annals of Allergy, Asthma & Immunology*	906
*World Allergy Organization Journal*	18	*Journal of Allergy and Clinical Immunology: In Practice*	711
*Journal of Dermatological Treatment*	17	*Clinical & Experimental Allergy*	657
*Annals of Allergy, Asthma & Immunology*	16	*New England Journal of Medicine*	653
*Journal of the European Academy of Dermatology and Venereology*	15	*Journal of the European Academy of Dermatology and Venereology*	573
*Frontiers in Immunology*	14	*Journal of the American Academy of Dermatology*	546
*International Archives of Allergy and Immunology*	14	*Journal of Investigative Dermatology*	398

The data in this table are corrected raw data and have not undergone journal name normalization processing.

On the basis of the Bradford law,^11^^,^^12^ it was determined that the number of core journals in the field was 10, and they accounted for 5.8% of all journals (10 of 171) (Fig 3, *E*). These core journals were consistent with the list of the top 10 journals listed, further demonstrating the importance of these 10 journals within the research field (Table III).

**Fig 4:**
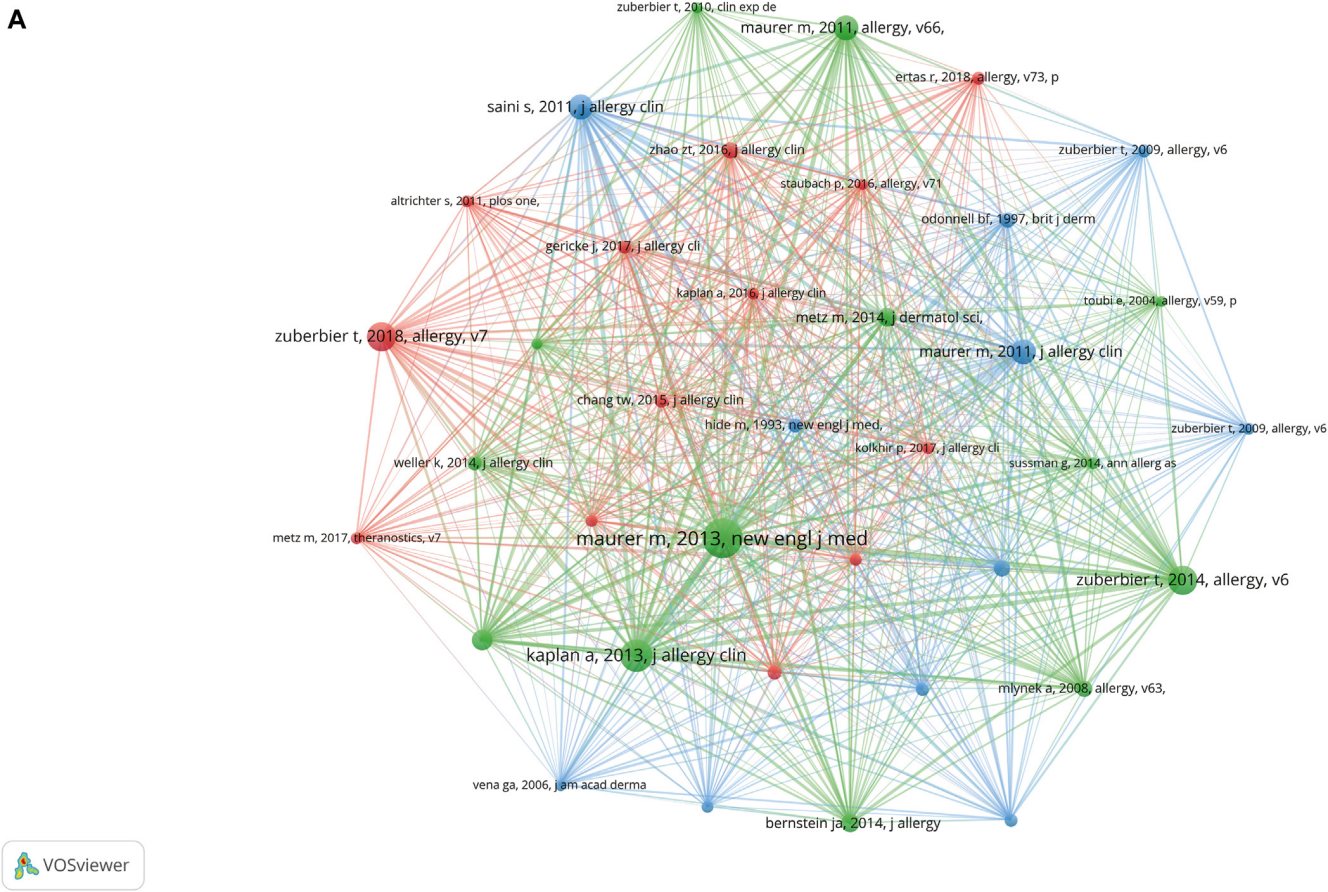

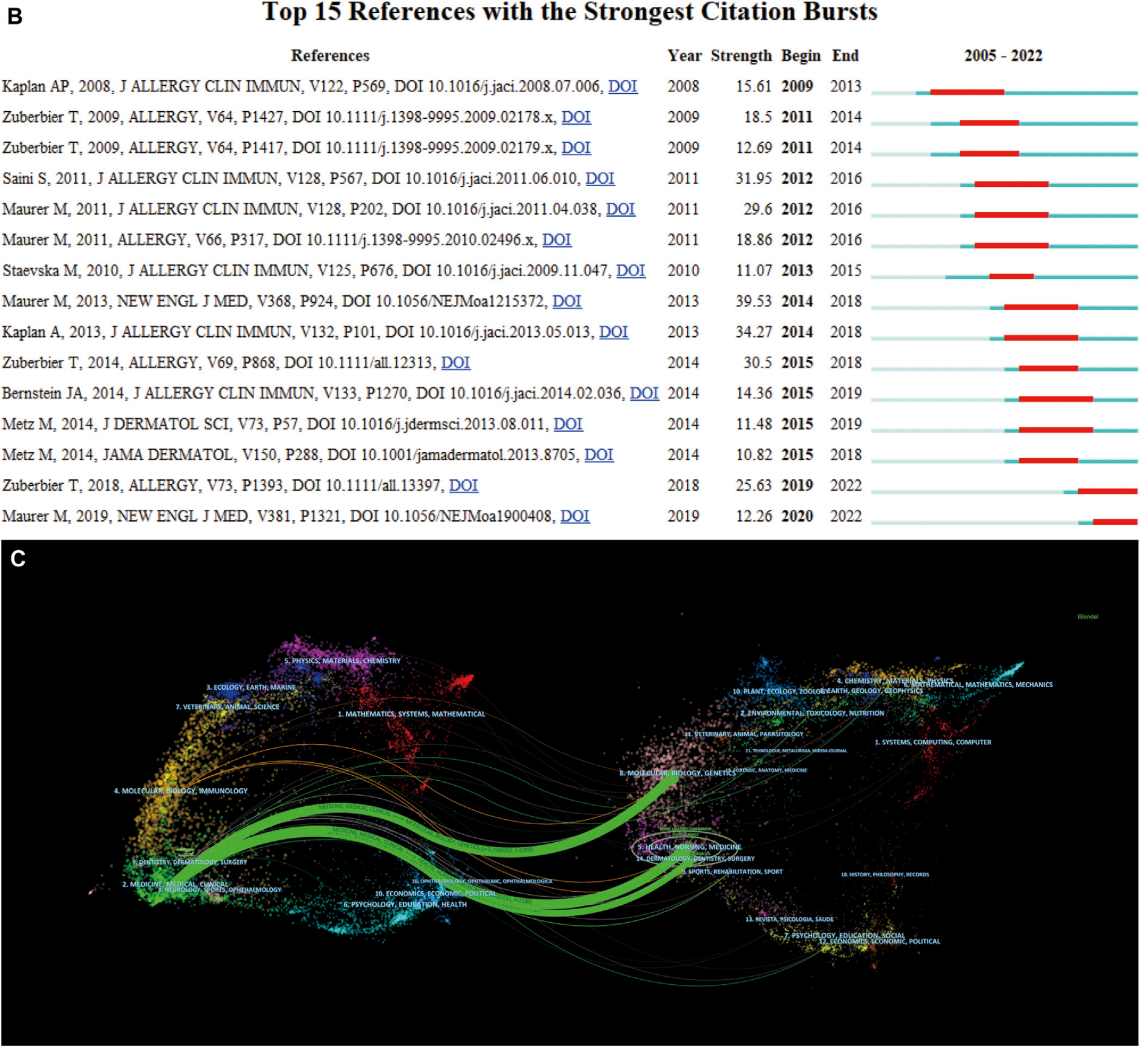
Dual-map overlay, top 15 citation bursts, and cocited references. **A,** The dual-map overlay of journals related to the research on omalizumab in CSU. The citing journals are on the left, and the cited journals are on the right. The label shows the research field of the journal cluster. Colored lines show the citation paths from left to right. Three different citation pathways were present. **(B)** Top 15 references with the strongest citation bursts. **(C)** Visualization of cocited references related to research on omalizumab in CSU.

The dual-map overlay of journals showed how the journals' topics were distributed (Fig 4, *A*). According to the 3 green citation channels, studies from the journals devoted to health, nursing, and medicine; dermatology, dentistry, and surgery; and molecular, biology, and genetics were frequently cited in articles from the journals devoted to medical topics, medicine, and clinical topics.

### Reference with citation bursts and cocited references

Over the past 2 decades, there have been 9780 cocited references related to research on use of omalizumab in CSU. The average number of cocitations in the top 15 cocited references was 130.3, with each cocited at least 69 times (Table IV). We used references with at least 50 cocitations to construct the cocitation network map (Fig 4, *C*).

**Table IV:** The top 15 cocited references

Cocited references	No. of citations
Maurer M, et al. New Engl J Med 2013;368:924. https://doi.org/10.1056/nejmoa1215372	262
Kaplan A, et al. J Allergy Clin Immunol 2013;132:101. https://doi.org/10.1016/j.jaci.2013.05.013	203
Zuberbier T, et al. Allergy 2018;73:1393. https://doi.org/10.1111/all.13397	176
Zuberbier T, et al. Allergy 2014;69:868. https://doi.org/10.1111/all.12313	174
Maurer M, et al. Allergy 2011;66:317. https://doi.org/10.1111/j.1398-9995.2010.02496.x	146
Saini S, et al. J Allergy Clin Immunol 2011;128:567. https://doi.org/10.1016/j.jaci.2011.06.010	144
Maurer M, et al. J Allergy Clin Immunol 2011;128:202. https://doi.org/10.1016/j.jaci.2011.04.038	141
Saini SS, et al. J Invest Dermatol 2015;135:67. https://doi.org/10.1038/jid.2014.306	112
Metz M, et al. J Dermatol Sci 2014;73:57. https://doi.org/10.1016/j.jdermsci.2013.08.011	102
Bernstein JA, et al. J Allergy Clin Immunol 2014;133:1270. https://doi.org/10.1016/j.jaci.2014.02.036	99
Mlynek A, et al. Allergy 2008;63:777. https://doi.org/10.1111/j.1398-9995.2008.01726.x	85
Zhao ZT, et al. J Allergy Clin Immunol 2016;137:1742. https://doi.org/10.1016/j.jaci.2015.12.1342	85
Kaplan AP, et al. J Allergy Clin Immunol 2008;122:569. https://doi.org/10.1016/j.jaci.2008.07.006	83
Odonnell BF, et al. Br J Dermatol 1997;136:197.	74
Kaplan AP, et al. Allergy 2017;72:519. https://doi.org/10.1111/all.13083	69

As shown in Table IV, the 2013 article titled “Omalizumab for the Treatment of Chronic Idiopathic or Spontaneous Urticaria” (Maurer M, et al. *N Engl J Med* 2013;368:924-35)^13^ has shown active cocitation relationships with other studies, such as “Omalizumab in Patients with Symptomatic Chronic Idiopathic/Urticaria Spontaneous despite Standard Combination Therapy” (Kaplan A, et al. *J Allergy Clin Immunol* 2013;132:101-9)^14^ and “The EAACI/GA(2)LEN/EDF/WAO Guideline for the Definition, Classification, Diagnosis, and Management of Urticaria: The 2013 Revision and Update” (Zuberbier T, *Allergy* 2014;69:868-87).^15^

In addition, we found that with regard to the top 15 cocited articles, the *Journal of Allergy and Clinical Immunology* published 6 (40%) *Allergy* published 5 (33%), and the remaining journals published 1 article each (Table IV). Clearly, the *Journal of Allergy and Clinical Immunology* and *Allergy* are central to the research on use of omalizumab in patients with CSU and are the major contributors to this field of research.

CiteSpace was used to assess the references with a high citation burst. As shown in Fig 4, *B* and Table V, we found that among the 15 most cited references, the article “Omalizumab for the Treatment of Chronic Idiopathic or Spontaneous Urticaria” (Maurer M, *N Engl J Med* 2013;368:924; 2014-2018 [the duration of the burst citation]; strength 39.55)^13^ was the article with the highest burst strength. The article with the second highest burst strength is “Omalizumab in Patients with Symptomatic Chronic Idiopathic/Spontaneous Urticaria despite Standard Combination Therapy” (Kaplan A, et al. *J Allergy Clin Immunol* 2013;132:101; 2014-2018; strength 34.28).^14^ The article with the third highest burst strength is “A Randomized, Placebo-Controlled, Dose-Ranging Study of Single-Dose Omalizumab in Patients with H1-Antihistamine–Refractory Chronic Idiopathic Urticaria (Saini S, et al. *J Allergy Clin Immunol* 2011;128:567; 2012-2016; strength 31.96]).^16^ We also found a high burst of citation intensity in recent years with the articles “Ligelizumab for Chronic Spontaneous Urticaria” (Maurer M et al. *N Engl J Med* 2019;381:1321; 2020-2022, strength 12.26)^17^ and “The EAACI/GA^2^LEN/EDF/WAO Guideline for the Definition, Classification, Diagnosis and Management of Urticaria” (Zuberbier T, et al. *Allergy* 2018;73:1393; 2019-2022; strength 25.66).^18^ They are all essential documents regarding the research on use of omalizumab in patients with CSU.

**Table V:** Representative burst citations in the top 15 articles with the most powerful citation burst

Begin	End	Strength	Year	Reference
2009	2013	15.6122	2008	Kaplan AP, et al. J Allergy Clin Immunol 2008;122:569. https://doi.org/10.1016/j.jaci.2008.07.006
2011	2014	18.5006	2009	Zuberbier T, et al. Allergy 2009;64:1427. https://doi.org/10.1111/j.1398-9995.2009.02178.x
2011	2014	12.6897	2009	Zuberbier T, et al. Allergy 2009;64:1417. https://doi.org/10.1111/j.1398-9995.2009.02179.x
2012	2016	31.9591	2011	Saini S, et al. J Allergy Clin Immunol 2011;128:567. https://doi.org/10.1016/j.jaci.2011.06.010
2012	2016	29.609	2011	Maurer M, et al. J Allergy Clin Immunol 2011;128:202. https://doi.org/10.1016/j.jaci.2011.04.038
2012	2016	18.868	2011	Maurer M, et al. Allergy 2011;66:317. https://doi.org/10.1111/j.1398-9995.2010.02496.x
2013	2015	11.0739	2010	Staevska M, et al. J Allergy Clin Immunol 2010;125:676. https://doi.org/10.1016/j.jaci.2009.11.047
2014	2018	39.5507	2013	Maurer M, et al. N Engl J Med 2013;368:924. https://doi.org/10.1056/NEJMoa1215372
2014	2018	34.2844	2013	Kaplan A, et al. J Allergy Clin Immunol 2013;132:101. https://doi.org/10.1016/j.jaci.2013.05.013
2015	2018	30.5197	2014	Zuberbier T, et al. Allergy 2014;69:868. https://doi.org/10.1111/all.12313
2015	2019	14.3691	2014	Bernstein JA, et al. J Allergy Clin Immunol 2014;133:1270. https://doi.org/10.1016/j.jaci.2014.02.036
2015	2019	11.4872	2014	Metz M, et al. J Dermatol Sci 2014;73:57. https://doi.org/10.1016/j.jdermsci.2013.08.011
2015	2018	10.82	2014	Metz M, et al. JAMA Dermatol 2014;150:288. https://doi.org/10.1001/jamadermatol.2013.8705
2019	2022	25.6559	2018	Zuberbier T, et al. Allergy 2018;73:1393. https://doi.org/10.1111/all.13397
2020	2022	12.2615	2019	Maurer M, et al. N Engl J Med 2019;381:1321. https://doi.org/10.1056/NEJMoa1900408

### Key words

**Fig 5:**
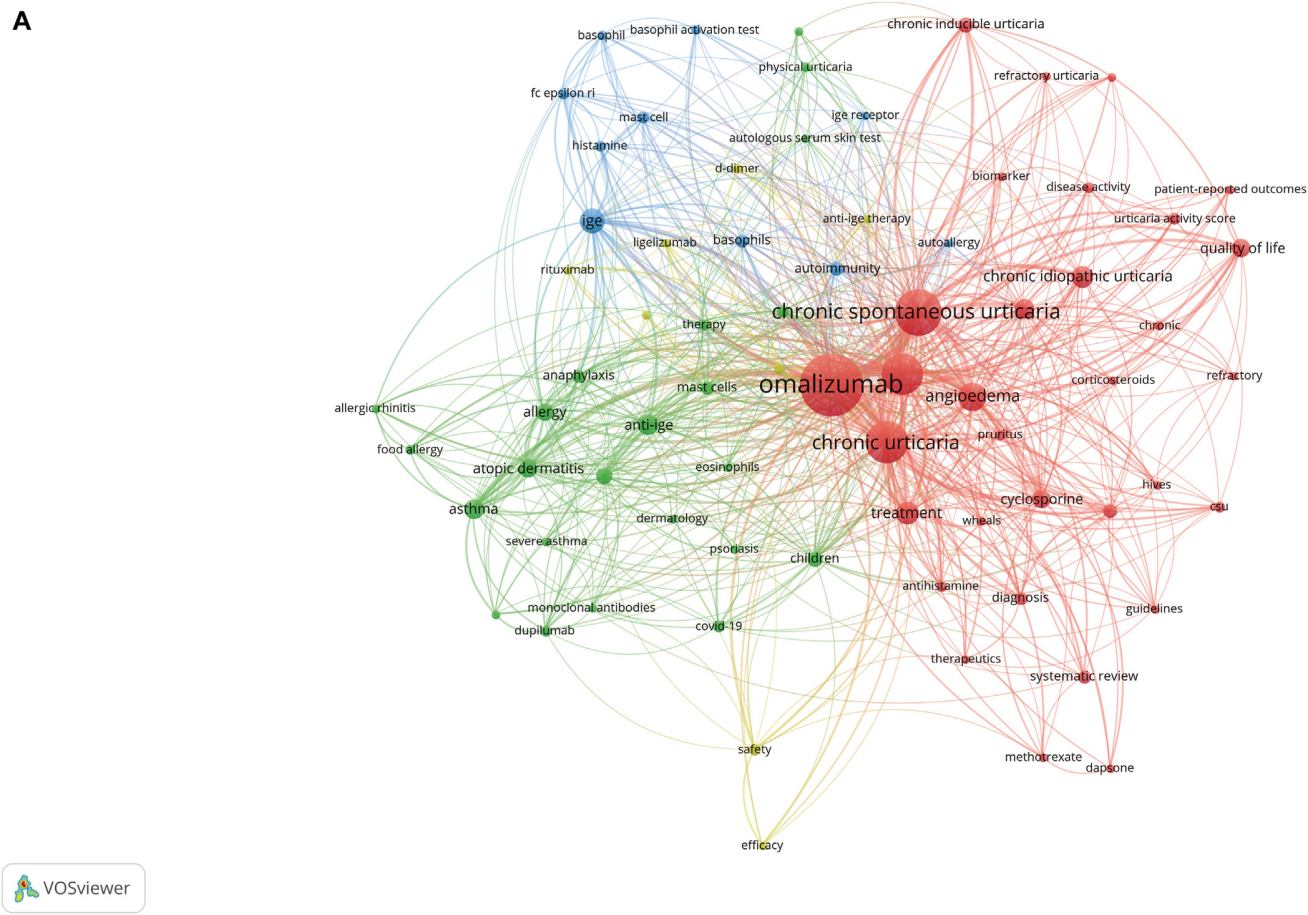

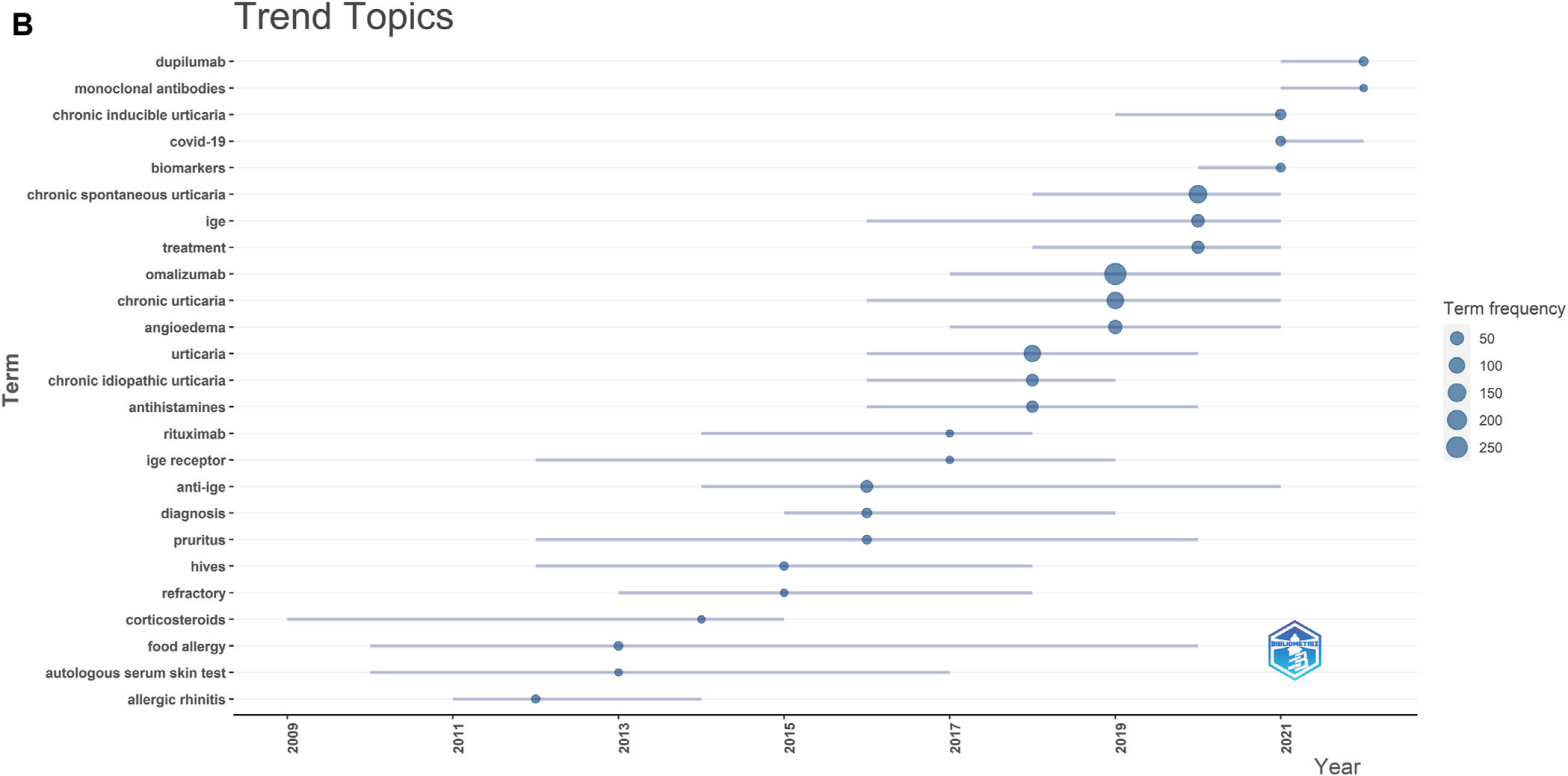
Key word co-occurrence network and trend topic analysis. **A,** Visualization of key word co-occurrence network of research on omalizumab in CSU. **B,** Trend topic analysis of research on omalizumab in CSU.

Using VOSviewer, from 862 author key words, we selected 73 key words with at least 5 occurrences to form the temporal co-occurrence network visualization (Fig 5, *A*). We set the resolution to 0.6 and generated 4 clusters, representing the 4 different research directions of research on use of omalizumab in CSU (Table VI). After excluding key search words and ambiguous words, we identified several key words with higher frequency; they included angioedema (n = 54), IgE (n = 42), treatment (n = 37), anti-IgE (n = 30), asthma (n = 28), atopic dermatitis (n = 25), and others.

**Table VI:** Temporal co-occurrence network visualization

Key words	Cluster	Occurrences
Safety	4	11
Biomarkers	4	8
D-Dimer	4	7
Anti-IgE therapy	4	6
Efficacy	4	6
Ligelizumab	4	6
mAb	4	5
Rituximab	4	5
IgE	3	42
Autoimmunity	3	15
Basophils	3	12
Mast cell	3	11
FcεRI	3	9
Histamine	3	8
Basophil	3	7
Basophil activation test	3	7
Autoallergy	3	6
IgE receptor	3	5
Anti-IgE	2	30
Asthma	2	28
Atopic dermatitis	2	25
Allergy	2	20
Biologics	2	19
Children	2	16
Anaphylaxis	2	13
Mast cells	2	12
COVID-19	2	11
IgE	2	10
Dupilumab	2	8
Therapy	2	8
Food allergy	2	7
Physical urticaria	2	7
Allergic rhinitis	2	6
Dermatology	2	6
Eosinophils	2	6
Mepolizumab	2	6
Psoriasis	2	6
Severe asthma	2	6
Autologous serum skin test	2	5
Cold urticaria	2	5
mAbs	2	5
Omalizumab	1	265
Chronic spontaneous urticaria	1	145
Chronic urticaria	1	119
Urticaria	1	118
Angioedema	1	54
Treatment	1	37
Chronic idiopathic urticaria	1	32
Antihistamines	1	27
Quality of life	1	25
Cyclosporine	1	24
Chronic inducible urticaria	1	16
Management	1	14
Systematic review	1	13
Diagnosis	1	12
Urticaria activity score	1	11
Antihistamine	1	9
CSU	1	9
Disease activity	1	8
Pruritus	1	8
Chronic	1	7
Guidelines	1	6
Hives	1	6
Patient-reported outcomes	1	6
Biomarker	1	5
Corticosteroids	1	5
Dapsone	1	5
Methotrexate	1	5
Refractory	1	5
Refractory urticaria	1	5
Therapeutics	1	5
Updosing	1	5
Wheals	1	5

In addition, we used the R package bibliometrix to conduct a trend topic analysis of the research on use of omalizumab in CSU. As shown in Fig 5, *B*, from 2012 to 2017, the research centered mainly on the diagnosis, treatment, and underlying mechanisms of CSU, with biologic agents playing a minor role. Since 2017 however, there has been a significant increase in attention devoted to biomarkers and the use of biologic agents, such as omalizumab and dupilumab, as a mainstream area of research for treating CSU. Moreover, recent years have seen a growing interest in the relationship between CSU and coronavirus 2019 (COVID-19). Table VII provides further insights into these trends.

**Table VII:** Recent research trend topics

Topic	Frequency	Year_Q1	Year_med	Year_Q3
Allergic rhinitis	6	2011	2012	2014
Food allergy	7	2010	2013	2020
Autologous serum skin test	5	2010	2013	2017
Corticosteroids	5	2009	2014	2015
Hives	6	2012	2015	2018
Refractory	5	2013	2015	2018
Anti-IgE	30	2014	2016	2021
Diagnosis	12	2015	2016	2019
Pruritus	8	2012	2016	2020
IgE receptor	5	2012	2017	2019
Rituximab	5	2014	2017	2018
Urticaria	118	2016	2018	2020
Chronic idiopathic urticaria	32	2016	2018	2019
Antihistamines	27	2016	2018	2020
Omalizumab	265	2017	2019	2021
Chronic urticaria	119	2016	2019	2021
Angioedema	54	2017	2019	2021
CSU	145	2018	2020	2021
IgE	42	2016	2020	2021
Treatment	37	2018	2020	2021
Chronic inducible urticaria	16	2019	2021	2021
COVID-19	11	2021	2021	2022
Biomarkers	8	2020	2021	2021
Dupilumab	8	2021	2022	2022
mAbs	5	2021	2022	2022

*Year_med,* Median year of publication of all documents, that is, 50% of the documents were published before this year.

## Discussion

### Summary

This study presents a comprehensive bibliometric review of research on omalizumab in patients with CSU over the past 20 years (2003-2022). Our analysis has revealed several significant findings. First, the United States and Charite University Medical Berlin were the most influential country and institution, respectively, with the highest numbers of articles and citations. Second, Marcus Maurer was this field's most published and cocited scholar. Third, the *Journal of Allergy and Clinical Immunology: In Practice* had the highest number of publications, whereas the *Journal of Allergy and Clinical Immunology* was the most cocited journal. Fourth, the diagnosis and treatment of CSU and its biologic mechanisms have long received extensive attention. Finally, the current research hotspots in this field were COVID-19, ligelizumab, dupilumab, and biomarkers. These findings provide valuable insights to help researchers understand the current state of research on omalizumab in CSU and identify potential research directions and hotspots in this field.

During period I (2003-2004), there was no relevant literature related to the research on omalizumab in CSU, indicating that relevant research had not been carried out at this stage. At this stage, omalizumab had just (in 2003) been approved by the US Food and Drug Administration (FDA) for treating patients aged 12 years and older with moderate-to-severe persistent allergic asthma,^19^ so off-label drug research was still in its infancy. In 2005, Mankad and Burks published an article titled “Omalizumab: Other Indications and Unanswered Questions,^20^ which launched the era of research on user of omalizumab in CSU. The article summarized the clinical trials of omalizumab in patients with allergic rhinitis and discussed the pathophysiologic mechanisms of the effects of omalizumab on allergic rhinitis. It also discussed the potential role of omalizumab in combination with specific allergen immunotherapy and other potential indications for omalizumab in IgE-mediated disorders, including food allergy, latex allergy, atopic dermatitis, and chronic urticaria. During period II (2005-2013), the research on omalizumab in CSU entered a new period. However, the number of publications in period II (which marked the initial stage of the research on omalizumab in CSU) was relatively small, with an average of about 5 publications annually. In period III (2014-2022), the number of publications began to increase significantly, with an average of about 57.9 annually.

The number of relevant publications published in 2014 was 29, which was 1.8 times the number in 2013. Also in 2014, omalizumab was approved by the FDA for treatment of chronic urticaria in patients in whom antihistamines were ineffective.^3^ In 2020, the number of publications on omalizumab in CSU reached 83. In period III, the number of publications on omalizumab in CSU showed an upward trend each year, reaching its peak in 2020. The total number of articles in this period increased significantly versus in the other 2 periods.

It is noteworthy that during our preliminary search, we determined that the database contained no relevant literature from the period spanning 1900 to 2002. Consequently, our research effectively comprises a comprehensive review of the entire developmental trajectory of the subject matter during this time frame.

### Major contributors and units

It was noted that the United States, Germany, Italy, and Spain dominated the field of study, which is also roughly in line with the distribution of author nationalities. The data showed that the United States had the most decisive influence in international research collaboration, particularly with Germany and Switzerland. Germany was also a significant collaborator, with a high frequency of collaboration with countries such as the United Kingdom, Switzerland, Spain, Turkey, Russia, and Canada. The United Kingdom had a vital role in international collaboration, with high frequencies of collaboration between the United Kingdom and Switzerland, Spain, Canada, Denmark, Poland, and Russia. The United States, Germany, United Kingdom, Spain, and Switzerland were the most influential countries in international research collaboration.

Regarding institutions, Charité Universitätsmedizin was ranked highest in terms of the number of publications and citations, and it should therefore be considered the institution with the most research strength and reputation in research on omalizumab in CSU. In recent years, the organization has focused extensively on such cutting-edge areas as the burden of CSU on patients and society,^21^ current and emerging treatments for CSU,^22^ evaluation of the effectiveness of therapies (including omalizumab in CSU treatment), and exploration of relevant biomarkers.^23^ Novartis Pharma AG and Novartis Pharmaceut are different components of the same company, and there is necessarily some collaboration between them. They both ranked high in terms of the number of publications. In recent years, the company has devoted attention to issues such as the association between severe acute respiratory syndrome coronavirus 2 (SARS-CoV-2) and CSU.^24^ Institutions such as Universitat Autònoma de Barcelona and the University of Toronto had many publications and citations. They influenced the field significantly and are an essential choice for researchers publishing in related fields. Of course, biotechnology companies, such as Genentech, collaborated less with other institutions but still had a high number of publications and citations, which indicates that the company was at the forefront of its research niche.

In terms of authors, Marcus Maurer is a leading core author in the field, having published 82 articles related to the research of omalizumab in CSU over the past 20 years. His first related article, “Efficacy and Safety of Omalizumab in Patients with Chronic Urticaria Who Exhibit IgE against Thyroperoxidase,”^25^ was published in 2011 and demonstrated the effectiveness of omalizumab in treating CSU. His research output has remained strong in the past 5 years, with about 45 articles published. Some of his highly cited articles include “Ligelizumab for Chronic Spontaneous Urticaria,”^17^ “Biomarkers and Clinical Characteristics of Autoimmune Chronic Spontaneous Urticaria: Results of the PURIST Study,”^26^, and “The Global Burden of Chronic Urticaria for the Patient and Society.”^21^ Marcus Maurer was not only involved in many areas of CSU but was also a core expert in the field of research on omalizumab in CSU.

At the same time, we found that Ana M. Gimenez-Arnau, Martin Metz, Petra Staubach, and other scholars have also made outstanding contributions. Ana M. Gimenez-Arnau is a scholar from the Universitat Autònoma de Barcelona. She is also involved in the study of omalizumab for the treatment of chronic idiopathic or spontaneous urticaria.”^13^ She is one of the pioneers in the field of research on omalizumab in CSU. Ana M. Gimenez-Arnau has remained committed to exploring this field in recent years. She has focused on the treatment of CSU in special populations, such as pregnant women^27^ and patients with COVID-19.^28^ At the same time, she also cooperated actively with other scholars to develop guidelines^29^ and explore new potential therapeutic approaches for CSU, including ligelizumab.^30^ Martin Metz is also a contributing scholar in this field. In recent years, he has focused on the deep mechanism of omalizumab in treating CSU^31^ and new treatments for CSU,^32^ including fenebrutinib^33^ and ligelizumab.^30^ Petra Staubach is a medical director at the clinical research center at the University of Mainz. In recent years, she has focused on the treatment of CSU in children and adolescents. Her main articles are “Severe Chronic Spontaneous Urticaria in Children—Treatment Options according to the Guidelines and Beyond—A 10-Year Review”^34^ and “Urticaria in Children and Adolescents: An Updated Review of the Pathogenesis and Management.”^35^

Notably, in the article titled “Ligelizumab for Chronic Spontaneous Urticaria,”^17^ the top 5 authors in terms of publication volume were actively involved in the study, which reflects the study's strong influence and significance and underscores its international and institutional collaborative nature, further highlighting the importance of global scientific cooperation in advancing medical knowledge.

By analyzing the status of publications by country, institution, and author, we can establish a preliminary understanding of the development of cooperation and research areas among different countries, institutions, and authors, which can inform further cooperation and research. Analysis of the relationship between authors and institutions aims to facilitate collaboration between experienced researchers and institutions to accelerate progress in this particular field.

### Popular journals and references

To assess the productivity and influence of journals in omalizumab research for CSU, we analyzed the number of published articles and cocitations. Over the past 2 decades, the *Journal of Allergy and Clinical Immunology: In Practice* had emerged as the most prolific publisher of articles (n = 34), whereas the *Journal of Allergy and Clinical Immunology* had garnered the highest number of cocitations (n = 4088), highlighting their significant contributions to the field. As official publications of the American Academy of Allergy, Asthma & Immunology, both journals have been recognized as authoritative sources. Notably, the *Journal of Allergy and Clinical Immunology: In Practice* is a sister journal of the *Journal of Allergy and Clinical Immunology*. Of the authors of the 34 articles published in The *Journal of Allergy and Clinical Immunology: In Practice*, Marcus Maurer was the most published author, with 8 articles representing 23.5% of the total. Strikingly, Marcus Maurer was also the author of 10 articles (40% of the total) in the *Journal of Allergy and Clinical Immunology*, the second most published (n = 25) and most cocited journal, suggesting a strong connection between the 2 journals.

We observed that the *Journal of Allergy and Clinical Immunology: In Practice* has recently published several articles on the management and treatment of CSU in different contexts, focusing on clinical practice. These include articles titled “Prevalence, Management, and Anaphylaxis Risk of Cold Urticaria: A Systematic Review and Meta-Analysis,”^36^ “Managing Chronic Urticaria and Recurrent Angioedema Differently with Advancing Age,”^37^ and “Biologics for the Use in Chronic Spontaneous Urticaria: When and Which,”^3^ among others.

In contrast, the *Journal of Allergy and Clinical Immunology* has published articles focusing predominantly on the mechanisms underlying CSU, with a bias toward basic research. These included articles titled “An Open-Label, Proof-of-Concept Study of Lirentelimab for Antihistamine-Resistant Chronic Spontaneous and Inducible Urticaria,”^38^ “The Efficacy of Omalizumab Treatment in Chronic Spontaneous Urticaria Is Associated with Basophil Phenotypes,”^29^ and “Response of Peripheral Blood Basophils in Subjects with Chronic Spontaneous Urticaria during Treatment with Omalizumab.”^39^

We also noted that although *Allergy* ranked fourth in terms of number of published articles (n = 22), it was second in terms of cocitation count (n = 2796). This indicated that although *Allergy* did not publish the most articles, the quality of its publications was generally high and they had a far-reaching impact. Its publications serve as a valuable reference for the research of omalizumab in CSU and have contributed significantly to the advancement of the field.

Through our analysis of academic fields of journals, we can conclude that articles from journals devoted to health, nursing, and medicine; dermatology, dentistry, and surgery; and molecular, biology, and genetics journals are frequently cited in journals in the medical, medicine, and clinical domain. This observation highlights the significance of cross-disciplinary collaboration and the potential for researchers in the medical, medicine, and clinical domain to expand and deepen their research by studying articles from related fields.

According to the analysis of the cocited literature, we obtained 3 clusters (Fig 4, *C*). We found that each cluster is different in terms of timing; that is, the 3 clusters showed the representative literature of the 3 periods. The blue cluster represented the earliest period of research, with the literature in the cluster having been published from 1993 to 2011. The earliest publication in this cluster was an article titled “Autoantibodies against the High-Affinity IgE Receptor as a Cause of Histamine Release in Chronic Urticaria,” which appeared in the *New England Journal of Medicine* in 1993.^40^ This groundbreaking article proposed that cross-linking of IgE receptors induced by autoantibodies may be a critical mechanism in the pathogenesis of chronic urticaria and other diseases mediated by mast cells. These new insights on the pathogenesis of CSU laid the foundation for subsequent research on related biologic agents. In 2011, Marcus Maurer and Sarbjit Saini published 2 pivotal studies in the *Journal of Allergy and Clinical Immunology*. The first study,^25^ conducted by Marcus Maurer, was a multicenter, randomized, double-blind, placebo-controlled trial aimed at assessing the efficacy of omalizumab treatment in patients with chronic urticaria who exhibited IgE antibodies directed against autoantigens, such as thyroperoxidase. The second study,^16^ conducted by Sarbjit Saini, was a prospective, double-blind, placebo-controlled, dose-ranging trial aimed at evaluating the efficacy and safety of omalizumab in patients with chronic idiopathic urticaria who remained symptomatic despite concomitant H1-antihistamine therapy. These 2 studies have demonstrated the efficacy and safety of omalizumab in treatment of chronic idiopathic urticaria, laying the foundation for subsequent research.

The earliest of the articles included in the green cluster was published in 2004, with the latest article published in 2015. Roughly, this period spans the years 2003 to 2014, when omalizumab was approved for treating moderate-to-severe allergic asthma in adults and children aged 12 years and older whose asthma was not controlled by inhaled steroids. In 2014, the FDA approved omalizumab for treating chronic idiopathic urticaria. Most of the articles within this cluster focused on using omalizumab to treat various allergic and immune-related conditions and explore its mechanisms of action and potential side effects. This period represented a significant milestone in development of omalizumab as a therapeutic agent and its application in various clinical settings. The core article in the green cluster is Marcus Maurer's study titled "Omalizumab for the Treatment of Chronic Idiopathic Urticaria: A Randomized Controlled Trial,”^13^ which was published in the *New England Journal of Medicine* in 2013. This phase 3, multicenter, randomized, double-blind study evaluated the efficacy and safety of omalizumab in patients with moderate-to-severe chronic idiopathic urticaria who remained symptomatic despite treatment with licensed doses of H1-antihistamines. The study demonstrated that omalizumab significantly reduced clinical symptoms and signs of chronic urticaria in patients who had not responded to approved doses of H1-antihistamines. This study provided strong evidence for the efficacy and safety of omalizumab in treating chronic idiopathic urticaria, which was critical in promoting its approval for clinical use. Most of the articles in the red cluster were published after 2014, with the pivotal article titled “The EAACI/GA^2^LEN/EDF/WAO Guideline for the Definition, Classification, Diagnosis, and Management of Urticaria,”^18^ which is a guideline published in 2018. This guide holds significant reference value for defining, classifying, diagnosing, and managing urticaria. It has also confirmed the effectiveness and safety of omalizumab in treating chronic idiopathic urticaria while providing relevant treatment recommendations.

To better predict the future research trends in this field, we examined the references with citation burstness. Among them, the article by Marcus Mauer titled “Omalizumab for the Treatment of Chronic Idiopathic or Spontaneous Urticaria”^13^ exhibited the highest burst strength, which aligns with the findings of cocitation relationship studies, further confirming the significance of this article in the research field. In addition, we will also note that Marcus Maurer’s appearance in many burst citation articles suggests that he has made significant contributions to the research in this field. His work has been widely recognized and cited by his peers, which suggests that his research may play a pivotal role in shaping future trends or directions in this field.

### General analysis of key words

Using key word clustering (Fig 5, *A*) and trend topic analysis, we have identified 4 distinct research directions for use of omalizumab in CSU and their corresponding development trends.

The red clustering nodes were the most numerous (n = 32), consisting of key words related to search conditions, and those without special significance. The main key words included angioedema, treatment, antihistamines, quality of life, cyclosporine, chronic inducible urticaria, management, urticaria activity score, and pruritus. Notably, search term–related phrases constituted the core portion of this clustering. Thus, this cluster focused primarily on CSU itself and emphasized clinical research. Because of the high number of key words within this cluster, we have rearranged them according to their meaning and identified 3 related subdivisions within this research direction. The first subdivision was the clinical diagnosis and evaluation of CSU. The representative key words were diagnosis, disease activity, and biomarker. The second subdivision direction was the study of management and treatment methods for clinical CSU. The core key words were therapeutics, corticosteroids, antihistamine, guidelines, management, and recurrence prevention. The third segmentation direction was patient quality of life, with the main key words being quality of life, patient-reported outcomes, and pruritus.

The second highest number of nodes (n = 23) was in the green clustering, with the main key words being anti-IgE, astroma, atopic dermatism, allergy, biology, children, anaphylaxis, mast cells, COVID-19, IgE, dupilumab, therapy, food allergy, and physical urticaria. This cluster included many other diseases, and the general research direction may be to explore the efficacy evaluation of use of omalizumab in treating CSU combined with diseases such as asthma, allergic dermatitis, and food allergy, as well as the application of other biologic agents such as dupilumab.

The main key words of the third-ranked cluster (n = 10) were IgE, autoimmunity, basophils, mast cell, FcεRI, histamine, basophil, basophil activation test, and autoallergy. These key words were centered on IgE. Therefore, the possible research direction was to explore the mechanism of IgE-mediated immune response and its regulation and further elucidate the specific mechanism, efficacy, safety, and long-term effects of omalizumab to treat related diseases such as CSU. It would involve experimental techniques such as IgE-mediated immune cells, CSU-related molecules, and basophil activation tests. Compared with the first cluster, this cluster was focused more on exploring basic experiments and more on studying biologic mechanisms.

The main key words in the last-ranked cluster (n = 8) were safety, biomarkers, D-dimer, anti-IgE therapy, efficacy, ligelizumab, mAb, and rituximab. These key words were centered on safety and biomarkers. Combined with other key words, this research direction was presumed to be focused mainly on new applications and safety of omalizumab and other biologics, as well as on exploration of reliable biomarkers to further explore the safety, mechanism of action, and mechanism of therapeutic evaluation of omalizumab in CSU.

In addition, our analysis reveals the presence of key terms such as *psoriasis, children, asthma, COVID-19*, and *food allergy,* among others. The frequencies of these key words varied. This observation highlights the great interest of researchers in exploring the diversity of clinical applications of omalizumab. In addition, researchers have conducted numerous studies on use of omalizumab in the pediatric population.

Overall, these clusters provide a comprehensive overview of the direction of research related to use of omalizumab in treatment of CSU, emphasizing clinical research, exploration of the application of biologic agents, study of biologic mechanisms, and exploration of safety and biomarkers. These findings can guide and inform future research related to CSU.

In addition, we conducted an analysis of the key word trend over time to identify changes in the research of omalizumab for the treatment of CSU. We have roughly divided the 25 trending topics into germination, development, and explosion periods according to time. The germination period was largely limited to the years 2005-2013. As early as 2005, Vaishali S. Mankad et al^20^ began focusing on and summarizing other indications for omalizumab, such as food allergy, latex allergy, atopic dermatitis, and (our study subject) CSU. Notably, it was clearly stated that omalizumab was an effective treatment for CSU. Also, Vaishali S. Mankad et al argued that the potential benefits of omalizumab need to be considered in the context of the cost of treatment and whether it is cost-effective when compared with the effects of treatment. Until 2013 however, the effectiveness of omalizumab was still somewhat controversial and its deeper mechanism of action remained poorly understood, with more exploratory studies and case data still needed.^41^ Since the FDA's 2014 approval of omalizumab for the treatment of CSU in adults and adolescents (aged ≥12 years) who remain symptomatic after H1-antihistamine therapy, research has shifted toward investigating topics such as anti-IgE, pruritus, and IgE receptors. The shift in research toward understanding the pathogenesis of CSU, understanding the mechanism of action of omalizumab therapy, and exploring potential treatment modalities beyond omalizumab reflected a growing interest in developing a deeper understanding of the condition. It also highlighted the fact that although omalizumab provided an effective treatment option, it was not a panacea for CSU. Indeed, although omalizumab, a humanized IgG-type anti-IgE antibody, is recommended for antihistamine-refractory cases, a significant proportion of patients still require third-line therapy, currently limited to cyclosporine, an immunosuppressant. The road to overcoming CSU is still long and requires continued research and investigation.^42^ The significant increase in research activity in the field from 2014 to 2017 underscored the urgent need to advance our understanding of the mechanisms underlying CSU and develop novel treatment approaches. Figure 1, *A* indicates that the number of articles related to CSU peaked from 2014 to 2017, with the main topics of discussion being mAbs, dupilumab, biomarkers, treatment, and COVID-19 (related to epidemics). Although several new treatments are developing, the underlying mechanisms of CSU and omalizumab treatment remain largely unknown. This highlights the need for continued research to improve our understanding of this disease and develop more effective treatment options.

Key word analysis helps simplify the preparation of researchers in the early stages of research. Identifying a large number of mAbs in key words not only marks the progress of mAb research within the CSU field at this stage but also allows researchers to anticipate drugs with great potential in this area.

### Treatment and mAbs

The current standard of care for CSU involves second-generation H1-antihistamines, with dose escalation in cases of partial or no response. Despite this approach, however, more than half of patients remain unresponsive to H1-antihistamine therapy,^43^^,^^44^ necessitating exploration of alternative treatment options. In response, novel therapies, such as mAbs and immunosuppressive agents (eg, cyclosporine, methotrexate, azathioprine, hydroxychloroquine), have been developed over the past 2 decades to improve symptom management and enhance patients' quality of life.^43^^,^^45^

At present, research on CSU therapy can be broadly classified according to the different pathways targeted: the IgE pathway (ligelizumab, UB-221, GI-310), the T2 pathway (dupilumab, benralizumab, mepolizumab, reslizumab), mast cell receptors (lirentelimab, CDX- 0159), complement (avdoralimab), and epithelial cytokines (tezepelumab).^46^ mAbs are biologic agents, and omalizumab is currently the only mAb approved for use in the treatment of CSU.^3^ However, ligelizumab is a promising mAb that is expected to be widely used in clinical practice shortly. It offers a new avenue for the expansion of biologic agents to treat CSU.^47^ Surprisingly, a recent experimental study showed that the dose-response curve for ligelizumab was apparent, with a response twice as high as that of omalizumab. It also had a faster response, better treatment effect, and longer duration than its counterpart omalizumab.^17^^,^^46^ However, phase 3 trials conducted on patients with CSU showed that specific test doses of ligelizumab demonstrated superiority over placebo controls but no significant difference when compared with omalizumab.^48^

Further research to determine the efficacy of ligelizumab is essential. In contrast, other anti-IgE drugs, such as UB-221 and GI-310, are still in the early development stages.^47^ Although dupilumab has advanced to phase 3 studies, the development of other targeted therapies is still in its infancy. Nonetheless, a second phase 3 trial of dupilumab in patients who are unresponsive to omalizumab was recently terminated owing to futility following a prespecified interim analysis.^48^ Therefore, it is expected that omalizumab will continue to be one of the main treatment alternatives for CSU soon. Moreover, additional investigations are necessary to enhance comprehension of various CSU subtypes and cellular immunologic mechanisms. These investigations will be indispensable in the quest for new CSU treatments.^47^

### Biomarkers

Although biomarkers of CSU have been studied for decades, no specific biomarker to diagnose CSU is available. Although biomarkers such as C-reactive protein, D-dimer, and total IgE do exist, they have not proved to be a “magic bullet” for diagnosing CSU.^23^^,^^49^ Recent research indicates that total IgE levels are a valuable marker for assessing disease activity, type, and treatment responses in patients with CSU. It is a reliable surrogate marker for distinguishing between type I and type IIb CSU.^50^ High total IgE levels may indicate disease activity and a positive response to omalizumab treatment, whereas low IgE levels may suggest type IIb autoimmune CSU and a better response to cyclosporine therapy.^46^ However, further studies are necessary to address crucial questions regarding the clinical differences in patients with different total IgE levels and the optimal cutoff value for low total IgE level.^50^

Meanwhile, the use of new mAbs and small molecules in CSU treatment may lead to more accurate patient stratification, better understanding of the disease's pathogenic mechanisms, and development of more sensitive and specific biomarkers.^49^ In conclusion, further research is necessary to better understand the properties and clinical usefulness of biomarkers in diagnosing and managing CSU. Specifically, studies are needed to identify biomarkers specific to CSU that can be used for diagnosing and monitoring disease progression. Moreover, it is essential to identify optimal treatments for individuals with CSU and to develop predictive biomarkers for treatment response.

### Advantages and shortcomings

Our study offers several distinctive advantages. First, although bibliometric studies on the topic of allergic skin disorders have been conducted previously,^51^ our study and this article represent the first comprehensive analysis of omalizumab research in the context of CSU. Furthermore, the study encompasses recent literature and utilizes diverse visualization methods, with the aim of offering inspiration to prospective researchers. Second, to ensure objectivity in our data analysis, we used 3 bibliometric tools—VOSviewer, Citespace, and the R package Bibliometrix—simultaneously. Additionally, compared with traditional reviews, our bibliometric approach offers a more comprehensive understanding of current trends and hotspots in the field.

Although our study has notable strengths, there are also some limitations that need to be addressed. First, the data sample size is relatively small, and only literature from the past 20 years was included, thus excluding data from before that time frame. Additionally, the study considers only data from the WoS database and disregards other literature databases. Second, there were some limitations during the data preprocessing stage, which resulted in some inaccuracies and inconsistencies in author and institutional names, leading to multiple nodes being occupied by the same person or institution in the graph. However, these incidents were rare and did not significantly affect the final results. Third, recently published high-quality articles may not have received sufficient attention owing to their low citation rate, thus highlighting the importance of future research updates. Despite these limitations, our study provides significant insights for academics and professionals seeking a better understanding of the studied fields.

However, bibliometric research holds undeniable significance within the domain of omalizumab and CSU. This research serves multiple essential purposes.

First and foremost, our research facilitates a systematic analysis and synthesis of pertinent literature in this field, allowing researchers across various disciplines to gain a comprehensive understanding of the field's origins, evolution, current status, and future prospects. Consequently, it offers valuable guidance and convenience to those who seek to investigate new issues, propose innovative methodologies, and generate fresh insights in this area. Furthermore, by using document relationship visualization techniques, this article enhances the clarity of information regarding researchers, research institutions, research outcomes, and other associated elements, thereby reducing the learning difficulty for new researchers. Additionally, the list of literature provided in this article serves as a valuable resource for those entering the field.

Secondly, bibliometric analysis empowers domain experts to develop a more holistic perception of research trends, consequently facilitating optimization of research strategies, resource allocation, and publication decisions. Thanks to tracking of the evolution of key words within the literature, this article anticipates significant research directions both presently and in the future.

Lastly, bibliometrics furnishes a quantitative benchmark for evaluating academic excellence within professional domains, thus contributing to the objectivity and equity of the academic evaluation system.

### Conclusions

This study provided a bibliometric and visual analysis of research on omalizumab in CSU over the past 20 years. The number of articles in this field has grown rapidly since 2014, with the United States, Germany, Italy, and Spain being the dominant countries. Charité Universitätsmedizin Berlin and Universitat Autònoma de Barcelona have been the leading research institutions, and Marcus Maurer, Ana M. Gimenez-Arnau, and Martin Metz have been the core experts in this field. Currently, the main research interests are focused on biomarkers and new therapeutic approaches for management of CSU, particularly mAbs. Therefore, future research should focus on understanding the pathogenesis of CSU; improving diagnosis, prognosis, and efficacy assessment at the biomarker level; and guiding development of new therapies, mainly biologics and immunosuppressive agents.

## Disclosure statement

Supported in part by the 10.13039/501100001809National Science Foundation of China (grant 82073434), Suzhou Science and 10.13039/100006180Technology Development Project (grant SKJYD202209), Suzhou Min sheng Technology-Medical and Health Application Foundation (grant SYS2020135), and 2020 Medical Research Project of Jiangsu Provincial Health Commission (grant Z2020017).

Disclosure of potential conflict of interest: The authors declare that have no relevant conflicts of interest.

## References

[1] Zuberbier T., Abdul Latiff A.H., Abuzakouk M., Aquilina S., Asero R., Baker D. (2022). The international EAACI/GA^2^LEN/EuroGuiDerm/APAAACI guideline for the definition, classification, diagnosis, and management of urticaria. Allergy.

[2] Fricke J., Ávila G., Keller T., Weller K., Lau S., Maurer M. (2020). Prevalence of chronic urticaria in children and adults across the globe: systematic review with meta-analysis. Allergy.

[3] Maurer M., Khan D.A., Elieh Ali Komi D., Kaplan A.P. (2021). Biologics for the use in chronic spontaneous urticaria: when and which. J Allergy Clin Immunol Pract.

[4] Kaplan A.P., Joseph K., Saini S.S. (2015). How omalizumab came to be studied as a therapy for chronic spontaneous/idiopathic urticaria. J Allergy Clin Immunol Pract.

[5] Ninkov A., Frank J.R., Maggio L.A. (2022). Bibliometrics: methods for studying academic publishing. Perspect Med Educ.

[6] Lewison G., Devey M.E. (1999). Bibliometric methods for the evaluation of arthritis research. Rheumatology.

[7] van Eck N.J., Waltman L. (2010). Software survey: VOSviewer, a computer program for bibliometric mapping. Scientometrics.

[8] Pan X., Yan E., Cui M., Hua W. (2018). Examining the usage, citation, and diffusion patterns of bibliometric mapping software: a comparative study of three tools. Journal of Informetrics.

[9] Synnestvedt M.B., Chen C., Holmes J.H. (2005). CiteSpace II: visualization and knowledge discovery in bibliographic databases. AMIA Annu Symp Proc.

[10] Aria M., Cuccurullo C. (2017). bibliometrix: an R-tool for comprehensive science mapping analysis. Journal of Informetrics.

[11] Yang J.M., Tseng S.F., Won Y.L., Juang J. (2016). Proceedings of the 3rd International Conference on Intelligent Technologies and Engineering Systems (ICITES2014). Lecture Notes in Electrical Engineering.

[12] Bradford S.C. (1934). Sources of information on specific subjects. Engineering.

[13] Maurer M., Rosén K., Hsieh H.J., Saini S., Grattan C., Gimenéz-Arnau A. (2013). omalizumab for the treatment of chronic idiopathic or spontaneous urticaria. N Engl J Med.

[14] Kaplan A., Ledford D., Ashby M., Canvin J., Zazzali J.L., Conner E. (2013). Omalizumab in patients with symptomatic chronic idiopathic/spontaneous urticaria despite standard combination therapy. J Allergy Clin Immunol.

[15] Zuberbier T., Aberer W., Asero R., Bindslev-Jensen C., Brzoza Z., Canonica G.W. (2014). The EAACI/GA^2^LEN/EDF/WAO guideline for the definition, classification, diagnosis, and management of urticaria: the 2013 revision and update. Allergy.

[16] Saini S., Rosen K.E., Hsieh H.J., Wong D.A., Conner E., Kaplan A. (2011). A randomized, placebo-controlled, dose-ranging study of single-dose omalizumab in patients with H1-antihistamine–refractory chronic idiopathic urticaria. J Allergy Clin Immunol.

[17] Maurer M., Giménez-Arnau A.M., Sussman G., Metz M., Baker D.R., Bauer A. (2019). Ligelizumab for chronic spontaneous urticaria. N Engl J Med.

[18] Zuberbier T., Aberer W., Asero R., Abdul Latiff A.H., Baker D., Ballmer-Weber B. (2018). The EAACI/GA^2^LEN/EDF/WAO guideline for the definition, classification, diagnosis and management of urticaria. Allergy.

[19] Humbert M., Busse W., Hanania N.A., Lowe P.J., Canvin J., Erpenbeck V.J. (2014). Omalizumab in asthma: an update on recent developments. J Allergy Clin Immunol Pract.

[20] Mankad V.S., Burks A.W. (2005). Omalizumab: other indications and unanswered questions. Clin Rev Allergy Immunol.

[21] Gonçalo M., Gimenéz-Arnau A., Al-Ahmad M., Ben-Shoshan M., Bernstein J.A., Ensina L.F. (2021). The global burden of chronic urticaria for the patient and society. Br J Dermatol.

[22] Johal K.J., Saini S.S. (2020). Current and emerging treatments for chronic spontaneous urticaria. Ann Allergy Asthma Immunol.

[23] Fok J.S., Kolkhir P., Church M.K., Maurer M. (2021). Predictors of treatment response in chronic spontaneous urticaria. Allergy.

[24] Bermingham W.H., Ardern-Jones M.R., Huissoon A.P., Krishna M.T. (2021). Forewarned is forearmed: chronic spontaneous urticaria as a potential risk to effective SARS-CoV-2 vaccine uptake and global public health. Br J Dermatol.

[25] Maurer M., Altrichter S., Bieber T., Biedermann T., Bräutigam M., Seyfried S. (2011). Efficacy and safety of omalizumab in patients with chronic urticaria who exhibit IgE against thyroperoxidase. J Allergy Clin Immunol.

[26] Schoepke N., Asero R., Ellrich A., Ferrer M., Gimenez-Arnau A., E H Grattan C. (2019). Biomarkers and clinical characteristics of autoimmune chronic spontaneous urticaria: results of the PURIST study. Allergy.

[27] Kocatürk E., Al-Ahmad M., Krause K., Gimenez-Arnau A.M., Thomsen S.F., Conlon N. (2023). Treatment patterns and outcomes in patients with chronic urticaria during pregnancy: results of PREG-CU, a UCARE study. J Eur Acad Dermatol Venereol.

[28] Kocatürk E., Salman A., Cherrez-Ojeda I., Criado P.R., Peter J., Comert-Ozer E. (2021). The global impact of the COVID-19 pandemic on the management and course of chronic urticaria. Allergy.

[29] Agache I., Rocha C., Pereira A., Song Y., Alonso-Coello P., Solà I. (2021). Efficacy and safety of treatment with omalizumab for chronic spontaneous urticaria: a systematic review for the EAACI Biologicals Guidelines. Allergy.

[30] Maurer M., Giménez-Arnau A., Bernstein J.A., Chu C.Y., Danilycheva I., Hide M. (2022). Sustained safety and efficacy of ligelizumab in patients with chronic spontaneous urticaria: a one-year extension study. Allergy.

[31] Metz M., Torene R., Kaiser S., Beste M.T., Staubach P., Bauer A. (2019). Omalizumab normalizes the gene expression signature of lesional skin in patients with chronic spontaneous urticaria: a randomized, double-blind, placebo-controlled study. Allergy.

[32] Eyerich S., Metz M., Bossios A., Eyerich K. (2020). New biological treatments for asthma and skin allergies. Allergy.

[33] Metz M., Sussman G., Gagnon R., Staubach P., Tanus T., Yang W.H. (2021). Fenebrutinib in H1 antihistamine-refractory chronic spontaneous urticaria: a randomized phase 2 trial. Nat Med.

[34] Staubach P., Peveling-Oberhag A., Lang B.M., Zimmer S., Sohn A., Mann C. (2022). Severe chronic spontaneous urticaria in children – treatment options according to the guidelines and beyond – a 10 years review. J Dermatolog Treat.

[35] Kudryavtseva A.V., Neskorodova K.A., Staubach P. (2019). Urticaria in children and adolescents: an updated review of the pathogenesis and management. Pediatr Allergy Immunol.

[36] Prosty C., Gabrielli S., Le M., Ensina L.F., Zhang X., Netchiporouk E. (2022). Prevalence, management, and anaphylaxis risk of cold urticaria: a systematic review and meta-analysis. J Allergy Clin Immunol Pract.

[37] Longhurst H.J., Gonçalo M., Godse K., Ensina L.F. (2021). Managing chronic urticaria and recurrent angioedema differently with advancing age. J Allergy Clin Immunol Pract.

[38] Altrichter S., Staubach P., Pasha M., Singh B., Chang A.T., Bernstein J.A. (2022). An open-label, proof-of-concept study of lirentelimab for antihistamine-resistant chronic spontaneous and inducible urticaria. J Allergy Clin Immunol.

[39] MacGlashan D., Saini S., Schroeder J.T. (2021). Response of peripheral blood basophils in subjects with chronic spontaneous urticaria during treatment with omalizumab. J Allergy Clin Immunol.

[40] Hide M., Francis D.M., Grattan C., Hakimi J., Kochan J.P., Greaves M.W. (1993). Autoantibodies against the high-affinity IgE receptor as a cause of histamine release in chronic urticaria. N Engl J Med.

[41] Babu K.S., Polosa R., Morjaria J.B. (2013). Anti-IgE – emerging opportunities for omalizumab. Expert Opin Biol Ther.

[42] Kolkhir P., Muñoz M., Asero R., Ferrer M., Kocatürk E., Metz M. (2022). Autoimmune chronic spontaneous urticaria. J Allergy Clin Immunol.

[43] Manti S., Giallongo A., Papale M., Parisi G.F., Leonardi S. (2022). Monoclonal antibodies in treating chronic spontaneous urticaria: new drugs for an old disease. J Clin Med.

[44] Kaplan A.P. (2017). Chronic spontaneous urticaria: pathogenesis and treatment considerations. Allergy Asthma Immunol Res.

[45] Lin W.K., Lin S.J., Lee W.R., Lin C.C., Lin W.C., Chang H.C. (2022). Effectiveness and safety of immunosuppressants and biological therapy for chronic spontaneous urticaria: a network meta-analysis. Biomedicines.

[46] Agache I., Akdis C.A., Akdis M., Brockow K., Chivato T., Del Giacco S. (2022). EAACI biologicals guidelines—omalizumab for the treatment of chronic spontaneous urticaria in adults and in the paediatric population 12-17 years old. Allergy.

[47] Wedi B. (2022). Emerging treatments for chronic urticaria. Expert Opin Investig Drugs.

[48] Kaplan A., Lebwohl M., Giménez-Arnau A.M., Hide M., Armstrong A.W., Maurer M. (2023). Chronic spontaneous urticaria: focus on pathophysiology to unlock treatment advances. Allergy.

[49] Asero R., Cugno M. (2021). Biomarkers of chronic spontaneous urticaria and their clinical implications. Expert Rev Clin Immunol.

[50] Altrichter S., Fok J.S., Jiao Q., Kolkhir P., Pyatilova P., Romero S.M. (2021). Total IgE as a marker for chronic spontaneous urticaria. Allergy Asthma Immunol Res.

[51] Podder I., Mondal H., Gayen R.K. (2023). Global research trend on allergic skin disorders: a bibliometric analysis from 2001 to 2020. Indian Dermatol Online J.

